# Understanding the Generation of Network Bursts by Adaptive Oscillatory Neurons

**DOI:** 10.3389/fnins.2018.00041

**Published:** 2018-02-06

**Authors:** Tanguy Fardet, Mathieu Ballandras, Samuel Bottani, Stéphane Métens, Pascal Monceau

**Affiliations:** ^1^Laboratoire Matière et Systèmes Complexes, UMR 7057, Université Paris Diderot, USPC, Paris, France; ^2^Department of Physics, Université d'Evry-Val d'Essonne, Évry, France

**Keywords:** bursting, adaptation, neuronal networks, synchrony, oscillators

## Abstract

Experimental and numerical studies have revealed that isolated populations of oscillatory neurons can spontaneously synchronize and generate periodic bursts involving the whole network. Such a behavior has notably been observed for cultured neurons in rodent's cortex or hippocampus. We show here that a sufficient condition for this network bursting is the presence of an excitatory population of oscillatory neurons which displays spike-driven adaptation. We provide an analytic model to analyze network bursts generated by coupled adaptive exponential integrate-and-fire neurons. We show that, for strong synaptic coupling, intrinsically tonic spiking neurons evolve to reach a synchronized intermittent bursting state. The presence of inhibitory neurons or plastic synapses can then modulate this dynamics in many ways but is not necessary for its appearance. Thanks to a simple self-consistent equation, our model gives an intuitive and semi-quantitative tool to understand the bursting behavior. Furthermore, it suggests that after-hyperpolarization currents are sufficient to explain bursting termination. Through a thorough mapping between the theoretical parameters and ion-channel properties, we discuss the biological mechanisms that could be involved and the relevance of the explored parameter-space. Such an insight enables us to propose experimentally-testable predictions regarding how blocking fast, medium or slow after-hyperpolarization channels would affect the firing rate and burst duration, as well as the interburst interval.

## Introduction

Network bursting is an intermittent collective behavior that occurs spontaneously in neuronal populations. It is characterized by long quiet periods, with almost no spike emission, punctuated by brief periods of intense spiking activity, where the whole network displays high firing rates—most neurons emit at least 2 closely-packed spikes. This particular pattern is then repeated, with varying regularity, over long time intervals.

Such periodic and synchronized activity has been observed as an emergent phenomenon in large neuronal populations, both in brain regions (Meister et al., 1991; Blankenship and Feller, 2009; Rybak et al., 2014) and unperturbed neuronal cultures (Wagenaar et al., 2006; Stegenga et al., 2008; Penn et al., 2016). It has been investigated as a plausible candidate for rhythmogenesis (Ramirez et al., 2004), but also in various disorders such as epilepsy (Derchansky et al., 2008) or Parkinson's disease.

Recent experiments by Penn et al. (2016), studying dissociated neuronal cultures where the chemical environment was precisely controlled, provide evidence that the majority of hippocampal pyramidal neurons are self-sustaining oscillators. These oscillators spontaneously synchronize to give birth to a very regular network bursting phenomenon.

Starting from these results and others (Ramirez et al., 2004; Suresh et al., 2016), we propose here a detailed understanding of the synchronized network bursting dynamics that explains and reproduces other experimental observations (Sipilä et al., 2006; Masquelier and Deco, 2013; Orlandi et al., 2013) of bursting on a variety of different timescales and with inter-burst intervals (IBIs) ranging from less than 1 s up to several minutes. We focus specifically on the characterization of the synchronized attractor and do not consider the transient synchronization process from an asynchronous to a synchronized phase. Indeed, synchronization of pulse-coupled oscillators is a known asymptotic behavior (Somers and Kopell, 1993; Bottani, 1995), which has been shown to lead to bursting in the presence of adaptation (Van Vreeswijk and Hansel, 2001). This was confirmed in all our simulations, regardless of the precise neuronal parameters, as long as they corresponded to adaptive oscillatory neurons. By oscillatory, we mean that a single neurons will spike periodically if uncoupled and considered independently.

Let us insist on the fact that collective bursting, giving rise to “network bursts,” should not be confused with the individual behavior observed at the cellular level for “bursting” or “chattering” neurons. Though they share similar intervals of rapid firing followed by long quiet periods (Connors and Gutnick, 1990; Sipilä et al., 2006), hence the common name, collective bursting can stem from radically different mechanisms and occur on different timescales (see Supplementary S1.3). Here, population-wide bursts are a specific synchronized behavior emerging from the interaction of oscillating, adaptive-spiking neurons which do not display intrinsic bursting behavior when considered independently but only emit single spikes.

The periodic activity of the intrinsically oscillatory neurons present in culture populations and brain regions is assumed to rely on leak currents which affect their excitability (Suresh et al., 2016). More specifically, the persistent, non-inactivating, sodium current *I*_*Na, p*_ (Golomb et al., 2006; Penn et al., 2016) and the H-current *I*_*h*_ (Lüthi and McCormick, 1998) are the prime candidates for this intrinsic depolarization. Adaptation, on the other hand refers to the capacity of a neuron to change—here, more precisely, to lower—its excitability in response to continuous or repeated excitation, such as a step-current in electrophysiological experiments, or the intense synaptic input received from its neighbors during a collective burst. Adaptive neurons indeed display periodic firing with a spiking frequency that progressively slows down from its initial high frequency value. The biophysical processes mediating adaptation are thus distinct for the origin of the rhythmic behavior which they modulate, and several potassium currents are considered for this frequency adaptation, like the muscarinic K^+^ (*I*_*M*_) current or the Ca^2+^ activated K^+^ currents (*I*_*AHP*_) (Sah and Louise Faber, 2002; Golomb et al., 2006).

We show here that adaptive spiking is a sufficient condition for network bursting, confirming what was suggested by previous studies (Van Vreeswijk and Hansel, 2001; Masquelier and Deco, 2013; Ferguson et al., 2015), and that intrinsically bursting or chattering neurons are not required. Indeed, we focus on the role of adaptation to explain why, as observed in the experiments, the presence of inhibitory neurons is not necessary to obtain regular collective bursting dynamics. Likewise, though short-term synaptic plasticity might play a role in shaping the dynamics (Gritsun et al., 2010; Masquelier and Deco, 2013), we also demonstrate that it is not required to reproduce characteristic timescales of this dynamics.

## Methods

We first describe the models used for the different units composing the system (neurons, synapses and network structure). Based on these, we derive an effective model which remains almost completely tractable, so that most of the properties of the collective dynamics can be predicted analytically. This model is based on successive approximations which were validated by numerical experiments: by dividing the cyclic behavior into several subdomains, we isolate regions where the activity can be solved under different approximations. The final solution is thus composed of the concatenation of these different approximations. We also used these simulations to verify and extend the predictions of our analytic equivalent model.

### Neuronal model

We chose the adaptive Exponential Integrate-and-Fire (aEIF) model (Brette and Gerstner, 2005) because of its compromise between simplicity and biological relevance. The dynamical evolution of a neuron is described by two variables, its membrane potential Ṽ, and a slow adaptation current $\tilde{w}$, which are governed by the following equations:

(1) $$\begin{array}{l} \text{if }\tilde{V}\leq {\tilde{V}}_{peak}\{\begin{array}{l} {\tilde{C}}_{m}\frac{d\tilde{V}}{d\tilde{t}}=-{\tilde{g}}_{L}(\tilde{V}-{\tilde{E}}_{L})+{\tilde{g}}_{L}{\tilde{\Delta }}_{T}e^{\frac{\tilde{V}-{\tilde{V}}_{th}}{{\tilde{\Delta }}_{T}}}\\ -\tilde{w}+{\tilde{I}}_{e}+{\tilde{I}}_{s}\\ {\tilde{\tau }}_{w}\frac{d\tilde{w}}{d\tilde{t}}=\tilde{a}(\tilde{V}-{\tilde{E}}_{L})-\tilde{w} \end{array}\\ \text{ else if }\tilde{V}>{\tilde{V}}_{peak},\text{ then }\{\begin{array}{ccc} \tilde{V} & \leftarrow & {\tilde{V}}_{r}\\ \tilde{w} & \leftarrow & \tilde{w}+\tilde{b} \end{array} \end{array}$$

where ${\tilde{C}}_{m}$ is the membrane capacitance, ${\tilde{g}}_{L}$ is the leak conductance of the neuron, *Ẽ*_*L*_ is its resting potential, $\tilde{\Delta }T$ affects both the slope and the strength of the spiking current, Ṽ_*th*_ is the threshold potential, ${\tilde{\tau }}_{w}$ is the adaptation timescale, ã gives the strength of subthreshold adaptation, $\tilde{b}$ gives the intensity of the spike-triggered adaptation, and Ṽ_*r*_ is the reset potential. Ṽ_*peak*_ is the spike cutoff for the model. Ĩ_*e*_ is an external current to which the neuron can be submitted.

The main difference of this model compared to the well-known integrate-and-fire model is the presence of the second variable, the current $\tilde{w}$, which modulates the neuronal excitability. The synaptic input received by a neuron is represented by the variable Ĩ_*s*_, which is usually time dependent. The neuronal adaptation can be either subthreshold, through the coupling between Ṽ and $\tilde{w}$ via ã, or spike-driven, from the step increments of size $\tilde{b}$ that $\tilde{w}$ undergoes after a spike.

The exponential spike generation present in the aEIF model is more realistic than the hard threshold of the original Integrate-and-Fire model, which leads to unrealistically fast spiking during bursts. The soft threshold of the Izikevich model (Izhikevich, 2007), which also includes adaptation and could have been a possible choice, is similar to that of the aEIF model and would be analytically more tractable. However, it generates a divergence which is not sharp enough, thus leading to overly long interspikes and induces an undesired influence of the cutoff value (*V*_*peak*_) on the neuronal dynamics (Touboul, 2009). Despite its non-analytic nature, this feature of the aEIF model was therefore critical to capture the inter-spike dynamics inside bursts.

In this study, and in accordance with the experimental observations for several types of pyramidal neurons, we use only neuronal parameters leading to adapting neurons which exhibit periodic spiking. This state is reached through the persistent current *Ĩ*_*e*_, which drives their progressive depolarization and makes them spike periodically; setting *V*_*r*_ < *V*_*th*_ ensures that the neurons are not intrinsically bursting, as described in Naud et al. (2008).

Contrary to the resting state, where one stable and one unstable fixed point exist (points where both $\dot{Ṽ}$ and $\dot{\tilde{w}}$ are zero), the periodic activity occurs after these two points disappear through a bifurcation, as described in Brette and Gerstner (2005) and Touboul and Brette (2008), when *I*_*e*_ becomes high enough. In this spiking regime, no fixed point is present in phase space, which allows the neuron to depolarize until *V*_*peak*_ before being reset to *V*_*r*_, thus following a discontinuous limit cycle.

Illustration of the resting and spiking behaviors can be found on Figures S2, S3, while biologically-relevant values of the parameters used for the aEIF model can be found in Table S1, in the Supplementary Material.

For these parameter sets, we have ${\tilde{\tau }}_{w}\gg {\tilde{\tau }}_{m}=\frac{{\tilde{C}}_{m}}{{\tilde{g}}_{L}}$, as the typical timescales for the continuous variation of $\tilde{w}$ relate to medium and slow after-hyperpolarization, which occur over hundreds of milliseconds (Sah and Louise Faber, 2002).

During the rest of the study, we use the dimensionless version of the model:

(2) $$\{\begin{array}{l} \dot{V}\;=-(V-E_{L})+e^{V}-w+I_{e}+I_{s}\\ \tau_{w}\dot{w}=a(V-E_{L})-w \end{array}$$

Details for the change of variables can be found in the first section of the Supplementary Material, “Neuronal model and parameters.” From then on, all equations involve only dimensionless variables and parameters.

### Synaptic model

The coupling strength between a pre-synaptic neuron *j* and a post-synaptic neuron *i*, such that *j* → *i*, is represented by the total charge *Q*_*s*_ transmitted from *j* to *i*. This charge is passed dynamically through the ion channels of the synapses, which we represent here by an alpha-shaped post-synaptic current (PSC) (Roth and van Rossum, 2009). If neuron *j* spikes at time *t*_*j*_, the triggered PSC is felt by *i*, after a delay *d*_*ji*_, and is described by:

(3) $$\begin{array}{l} I_{s}(t)=\Theta (t-t_{j}-d_{ji})I_{ji}(t-t_{j}-d_{ji})\\ \;=\;s_{ji}I_{0}\cdot (t-t_{j}-d_{ji})\Theta (t-t_{j}-d_{ji})e^{-\frac{t-t_{j}-d_{ji}}{\tau_{s}}}. \end{array}$$

Where *s*_*ji*_ is the strength of the synaptic connection from *j* to *i*, τ_*s*_ is the characteristic synaptic time, Θ(*x*) is the Heaviside step function, such that Θ(*x*) = 0 is *x* ≤ 0 and Θ(*x*) = 1 if *x* > 1, and $I_{0}=\frac{1pA}{{\tilde{g}}_{L}{\tilde{\Delta }}_{T}}$ is the unit current which we set in this way to be coherent with the conventions of the NEST simulator (Kunkel et al., 2017). As such, the total charge delivered to *i* reads:

(4) $$Q_{s,ji}=\int_{0}^{\infty }I_{ji}(t)dt=s_{ji}I_{0}\tau_{s}^{2}.$$

### Network models

This study is based on two non-spatial random network models: a fully homogeneous network with fixed in-degree which is useful to introduce the equivalent model, and more heterogeneous Gaussian in-degree networks which are supposed to be representative of connectivity in dissociated cultures (Cohen et al., 2010). Both random networks are generated in the same way by drawing a number *k*_*i*_ (in-degree) of incoming connections originating from randomly chosen other neurons in the population. In the case of fixed in-degree networks, the in-degree *k*_*i*_ is fixed and identical for each neuron. For Gaussian random networks, *k*_*i*_ is drawn for each neuron from a Gaussian distribution with mean value $\bar{k}$ and standard deviation σ_*k*_. Note that the fixed-in-degree networks can be seen as the limit case of the Gaussian ones when the variance goes to zero. The out-degree distributions are binomial and identical in both cases. All networks where generated using the graph-tool or igraph backends of the NNGT library.

All transmissions between neurons in the network are subjected to the same delay *d* and have the same synaptic strength *s*. which means that the complete dynamical system describing the network is given, for each neuron *i*, by:

(5) $$\{\begin{array}{l} {\dot{V}}_{i}\;=-(V_{i}-E_{L})+e^{V_{i}}-w_{i}+I_{e}+\underset{I_{syn,i}(t)}{\underset{︸}{\sum_{j\to i}\sum_{t_{j}}sI_{0}\cdot (t-t_{j}-d)\Theta (t-t_{j}-d)e^{-\frac{t-t_{j}-d}{\tau_{s}}}}}\\ \tau_{w}{\dot{w}}_{i}=a(V_{i}-E_{L})-w_{i} \end{array}$$

Where {*j* → *i*} is the set of neurons *j* that are presynaptic neurons for *i* and {*t*_*j*_} is the set of spike times for neuron *j*.

### Numerical simulations

All dynamical simulations were performed using the NEST simulator (Kunkel et al., 2017) with the aeif_psc_alpha model implementation, that we developed, and which corresponds to the Equations (2, 3) presented above. Neurons were set to adaptive spiking using the neuronal and synaptic parameters detailed in Table S1 and were connected using static_synapses, i.e., without plasticity, but including a delay *d* in the spike transmission. Simulations were started from a population of neurons in an asynchronous random state, with their state variable $\tilde{w}$ following a normal distribution of average value 50 pA and standard deviation 10 pA. The runs were performed on networks containing 1,000–100,000 neurons with an average degree of 100, which is the typical value estimated in mature neuronal cultures (Cohen et al., 2010).

### Activity analysis

For each simulation we computed the average firing rate $\nu =\frac{N_{s}}{T}$, where *N*_*s*_ is the total number of spike and *T* is the simulation time. This gives us a characteristic timescale *t*_ν_, which would be the average interspike if the spikes were distributed uniformly. Considering *d* as the transmission delay of action potentials, bursts are identified as uninterrupted sequences of spikes separated by less than min(*t*_ν_/2, 3*d*); they must also involve at least 20% of the neurons. This analysis was performed using tools from the NNGT library and extra functions available on our GitHub repository.

### Equivalent analytical model

**Figure 1:**
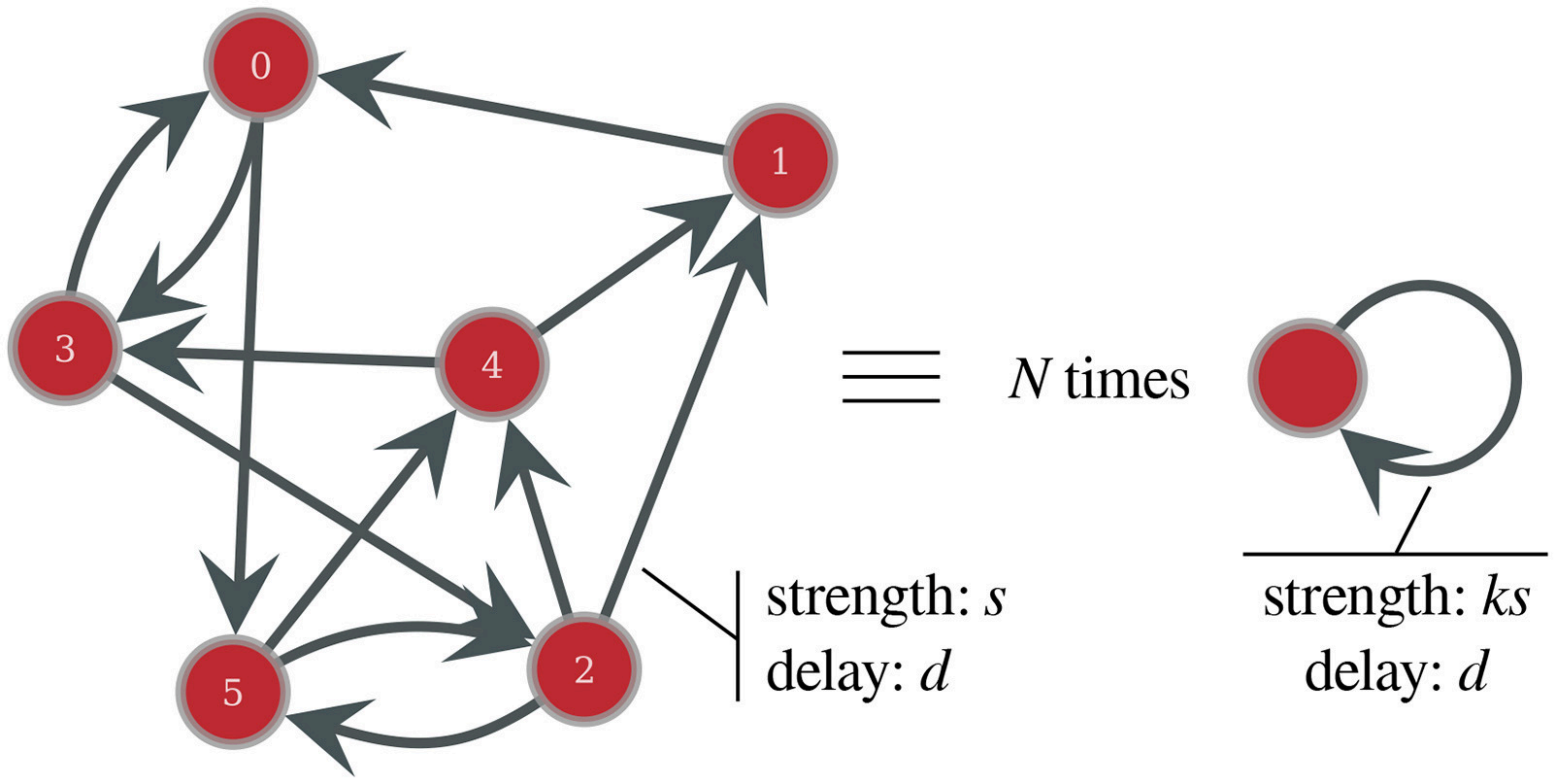
Schematic representation of the equivalence between a fixed-in-degree network containing *N* = 6 synchronous neurons, with in-degree *k* = 2 and connection strength *s*, and *N* isolated neurons, with a self-loop connection of strength *ks*.

We derived an equivalent model that describes the system dynamics and predicts the range over which the characteristic frequencies can vary without the need to simulate the network dynamics.The model focuses on the fully synchronized dynamics, for which all neurons behave almost identically. The rationale of the model is most apparent if we first consider the case of a fixed in-degree network. As illustrated on Figure 1, in this case, once the population is synchronized, all neurons receive the same input, that is the contribution of *k* simultaneous spikes given by the sum of *k* PSCs. Here, one neuron behaves exactly as any other neuron, thus, ∀*i, j, t V*_*i*_(*t*) = *V*_*j*_(*t*) = *V*(*t*).

This means that the network of *N* neurons receiving *k* inputs of strength *s* is equivalent to *N* isolated neurons, each one forming a close loop with one autapse—that is, a self-loop—of strength *k* × *s*. This simplification is inexact if all neurons do not have the same number of incoming connections, however, as shown in the Results section, this approximation holds very well for homogeneous Gaussian networks and, through a slight modification of the synaptic dynamics, even the behavior of more heterogeneous Gaussian or scale-free networks can be estimated.

Based on this observation, exact for fixed in-degree networks, we propose a model of bursting dynamics for any synchronized network, where we describe the whole population through the behavior of an equivalent neuron, representative of the “average” dynamics. This neuron is subjected to the “average” input received by neurons in the network, and, under this simplified description, Equation (6) is now the same for every neuron in the network, since they are all approximated by this equivalent neuron. As they all receive the same number of spikes (*n*_*s*_) emitted at the same times {*t*_*j*_}, *j* ∈ [1, …, *n*_*s*_], and from the same number *k* of neighbors, we obtain:

(6) $$\{\begin{array}{l} \dot{V}\;=-(V-E_{L})+e^{V}-w+I_{e}+\underset{I_{syn}(t)}{\underset{︸}{\sum_{j}ksI_{0}\cdot (t-t_{j}-d)\Theta (t-t_{j}-d)e^{-\frac{t-t_{j}-d}{\tau_{s}}}}}\\ \tau_{w}\dot{w}=a(V-E_{L})-w \end{array}$$

This single dynamical system is then solved through several approximations depending on the network state. A typical approximation in the burst, on the interval [*t*_*i*_, *t*_*i*_ + *d*] between the emission of a spike and its arrival, consists in linearizing the exponential term when *V* < *V*_*th*_. On this interval, *I*_*syn*_ = 0 and since *d* ≪ τ_*w*_, *w* can be considered as constant. This leads to an approximate solution for *V*(*t*) that we will call *V*_*l*_(*t*), with *V*_*l*_(*t*_*i*_) = *V*_*r*_ (see also Equations S2, S3 in Supplementary Material):

(7) $$V_{l}(t)=V_{r}e^{-t}+(E_{L}+I_{e}-w)\left(1-e^{-t}\right)\text{ for }t_{i}\leq t<t_{i}+d.$$

For *V* ∈ [*V*_*th*_, *V*_*peak*_], we cannot solve the equation, but know from simulations that this simply leads the neuron to spike with a typical timescale of τ_*m*_ = 1.

From these analytic formula, we can then constrain the final solution through a self-consistent equation. The solution of the self-consistent equation will therefore assure that the spikes of one neuron during a burst sustain the burst itself and drive the subsequent ones (self-loop in the equivalent representation of Figure 1), thus shaping a permanent and self-sustained bursting activity, as observed experimentally. Such a solution gives a complete description of the neuron's dynamical properties in time and allows us to obtain all the characteristics of the bursting dynamics.

This equivalent approach is applied here to three different synaptic models (instantaneous, continuous, and alpha-shaped synapses) leading to three transcendental self-consistent equations; details of mathematical developments can be found in the Supplementary Material. Python tools to solve the self-consistent equations and compute the characteristics of the bursting behavior are available on our GitHub repository; they are based on the scipy implementation of Brent's root-finding method.

### Exploration of parameter space

Thanks to the fast computation of the equivalent model, we were able to compute the dynamical properties for a large number of parameter sets. These results were normalized and analyzed through a Principal Component Analysis algorithm, using the scikit.learn package, in order to obtain the correlation matrix linking the collective dynamical properties to the precise values of the neuronal parameters.

For each parameter set, we first ensure that there is no stable fixed-point in phase-space and that the model predicts a solution, i.e., the existence of bursts with mathematically coherent properties. Secondly, we assess the biological relevance of the solution by (1) ruling out dynamics for which the voltage decreases to values lower than −120 mV during the giant hyperpolarization following a burst; (2) restricting the maximum value of the slow current *w* to 1,000 pA; (3) preventing cellular bursting for individual neurons by asserting *V*_*r*_ < *V*_*th*_—this restricts the neurons to single-spike intrinsic behaviors (Naud et al., 2008).

These constraints limit the number of “valid” parameter sets and make the parameters inter-dependent; this leads to a non-trivial parameter/parameter correlation matrix (Figure S1).

## Results

As mentioned in the introduction and discussed in the Supplementary Material, synchronization is highly resilient and we focus here solely on the fully synchronized bursting network. We start from individual neurons which are spiking periodically, a behavior that seems to originate from persistent sodium currents like *I*_*Na, p*_ or *I*_*h*_ in neuronal cultures (Penn et al., 2016); it is modeled here by a constant input current *I*_*e*_. When these neurons are coupled, however, their periodic dynamics is drastically modified as they adopt a collective bursting behavior (Borges et al., 2017).

We describe the attractor characterizing the dynamics of the synchronous bursting state. Our key result details the properties of this attractor and shows how they are linked to both the biological parameters of the neurons and the network topology.

The behavior shows features of a relaxation oscillator (see **Figure 4A**): the current *w* slowly decreases during the quiescent phase, then rapidly increases during the bursting phase until it reaches a threshold value w^*^, which determines the burst termination and the start of a new cycle.

The main characteristic which determines the dynamics is the maximum value of the adaptation current, w^*^ reached at the end of a burst. It depends on the neuronal and network parameters, and qualitatively obeys the following equation (details in subsection 5.3 of the Supplementary Material):

(8) $$w^{*}\approx E_{L}+I_{e}+\frac{V_{r}e^{-d}+\bar{k}Q_{s}}{1-e^{-d}}+C$$

where *C* is a constant. Since the firing rate during the burst is mostly linked to w^*^, this equation directly shows that higher coupling ($\bar{k}Q_{s}$), higher excitability (*E*_*L*_), or higher reset voltage (*V*_*r*_) will increase the bursting intensity. The effect of the transmission delay *d* is slightly more complex but roughly decreases bursting intensity when increased.

Taking into account finer effects and spike-driven adaptation then leads to more complete equations delivering additional results about the influence of the remaining parameters. These are considered in more details in the Discussion section.

In the following subsections, we describe and explain the bursting dynamics, then discuss the more detailed, self-consistent versions of Equation (9) (complete derivation of these equations can be found in the Supplementary Material). Finally, we describe how our model accounts for the structural heterogeneity that is present in neuronal cultures.

### The attractor, inner structure of a burst

**Figure 2:**
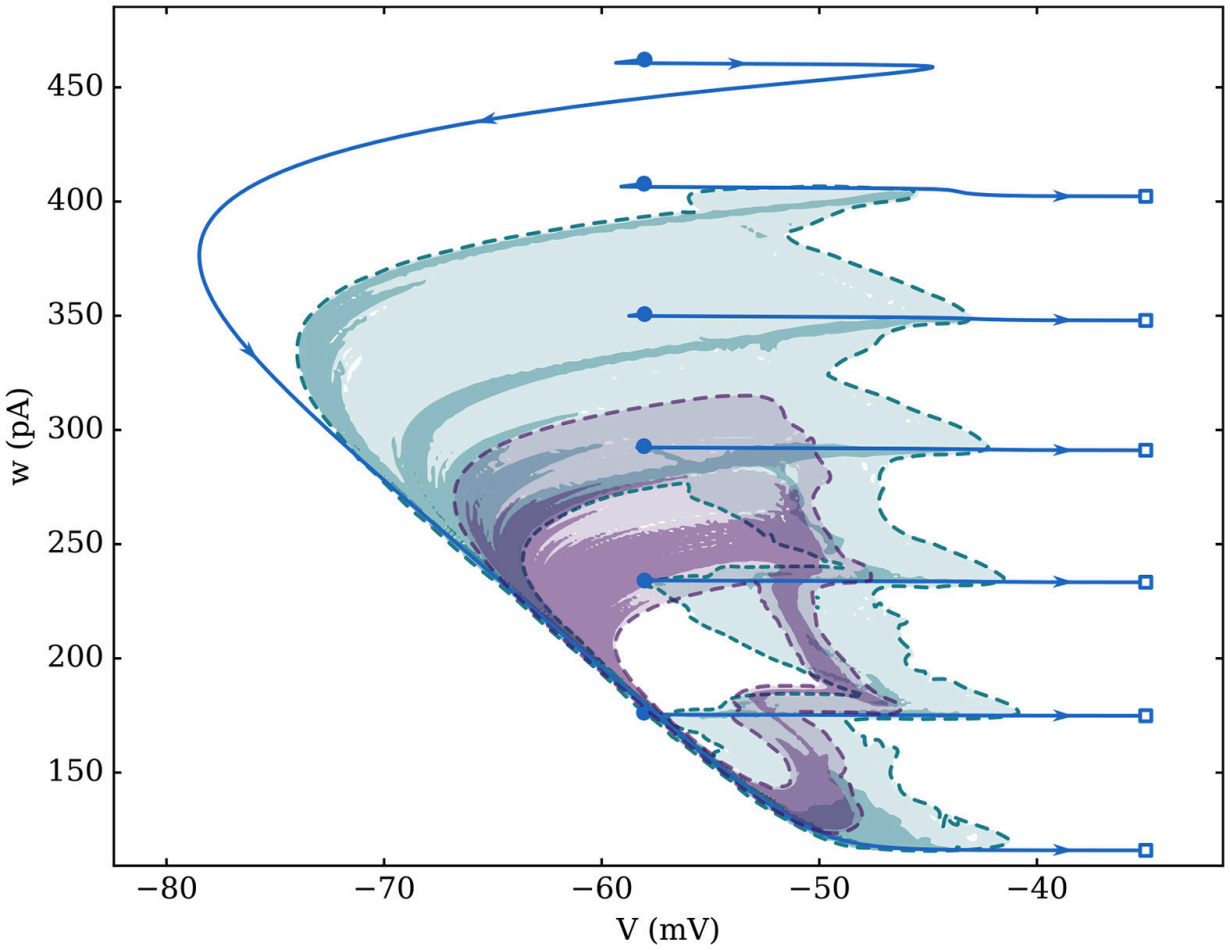
Attractors for three different networks of 1,000 identical neurons with average degree 100. Fixed-in-degree is represented by the blue solid line (spike positions are represented by empty squares and reset positions by full circles). For Gaussian in-degree networks, the logarithm of the number of states per bin—over 200 simulations with 4 cycles each—was used to compensate the non-constant velocity across the whole attractor. The larger attractor, in green, is associated to σ_*k*_ = 4; the smaller one, in purple, is for σ_*k*_ = 20; both attractors are delimited by a dashed line (limit of a unique visit per bin). Bin size is approximately 0.05 mV along the *V*-axis and 1 pA along the *w*-axis.

The synchronous attractor is composed of intermittent bursts of activity, as shown in Figure 2 in the (*V, w*) phase space. During a cycle, the neuron state variables (*V*, *w*) do not follow the attractor at constant velocity: the neuron spends much longer on the recovery path (low *V*) compared to the bursting period (high *V*)—see **Figure 4** to see this trajectory in time.

This attractor is modified by the presence of heterogeneity in the network's topology—quantified by σ_*k*_ for Gaussian in-degree networks—which impacts both its duration and regularity. Indeed, heterogeneity noticeably smooths the average behavior and reduces the number of spikes in a burst which goes down from 6 spikes per burst for the fixed in-degree graph, to 3–5 if σ_*k*_ = 4, and is roughly reduced to 2 when σ_*k*_ = 20. For the fully synchronized fixed in-degree network, all neurons are responding to the exact same input—they receive spikes from the same number of neighbors—hence they are all equivalent to a single average neuron.

**Figure 3:**
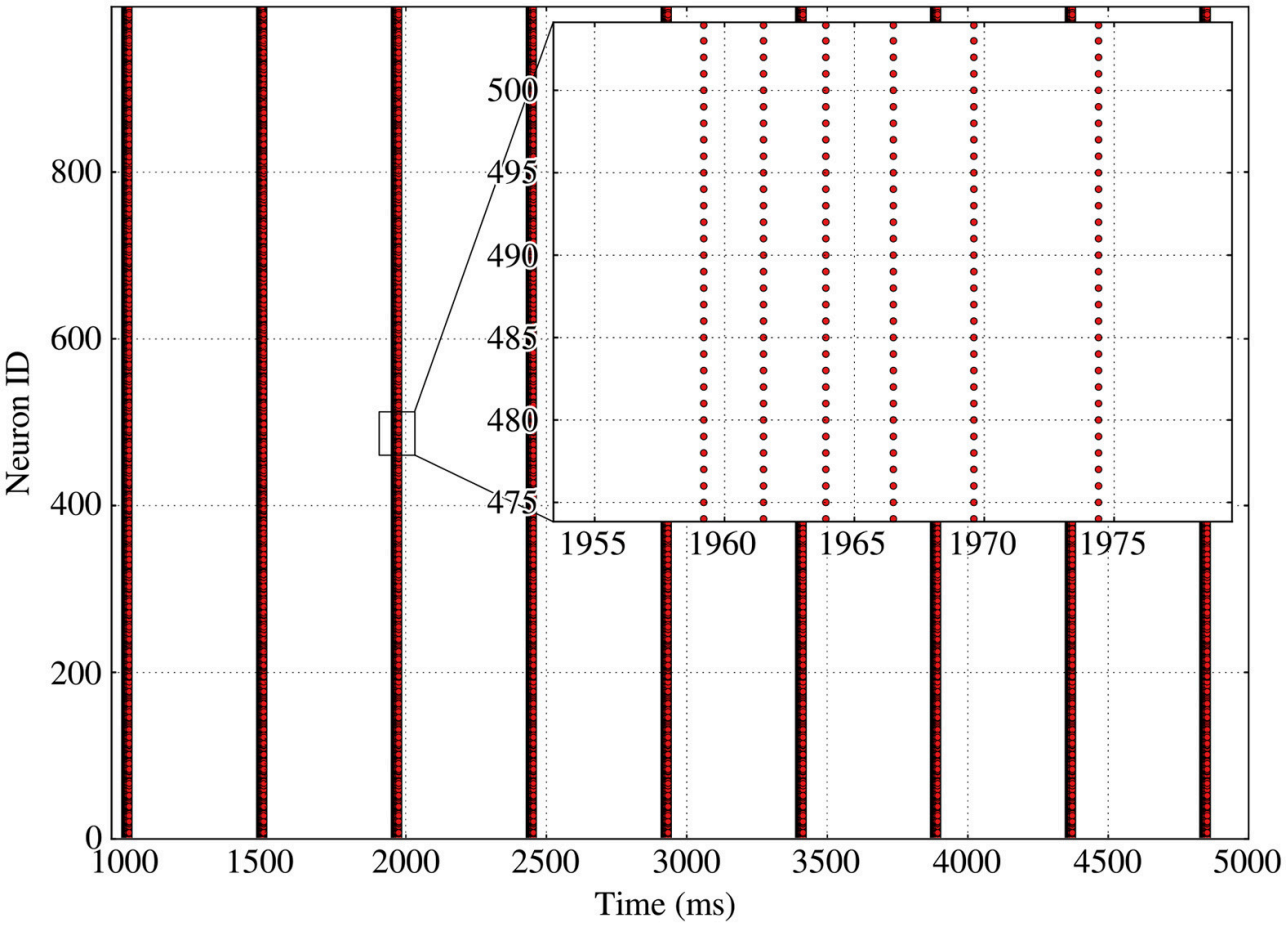
Spike raster of bursting activity for a fixed 100-in-degree network. Inset provides details on the behavior of the neurons during a single burst, with successive synchronized burst slices separated by longer and longer intervals as the adaptation increases.

As can be seen on Figure 3 for a fixed in-degree network, synchronized bursting of the population consists of a succession of active periods, called *bursts*^1^, separated by long inactive intervals, which we call recovery periods. As can be seen on the inset, the burst displays a strongly ordered inner structure composed of successive *synchronized burst slices*, which are consistent sets of spikes stemming from a common input.

This inner structure, based on spike events, helps us define several quantities that characterize the dynamics such as the burst and inter-burst durations. However, information about the spike times alone is not sufficient to provide insights regarding the phenomena involved in the burst initiation or termination. Therefore, we will use the time evolution of the neuron's state variables to perform a phase-plane analysis and investigate possible mechanisms for both the bursting and recovery periods.

### Neuronal trajectory, assessment of the theoretical model

**Figure 4:**
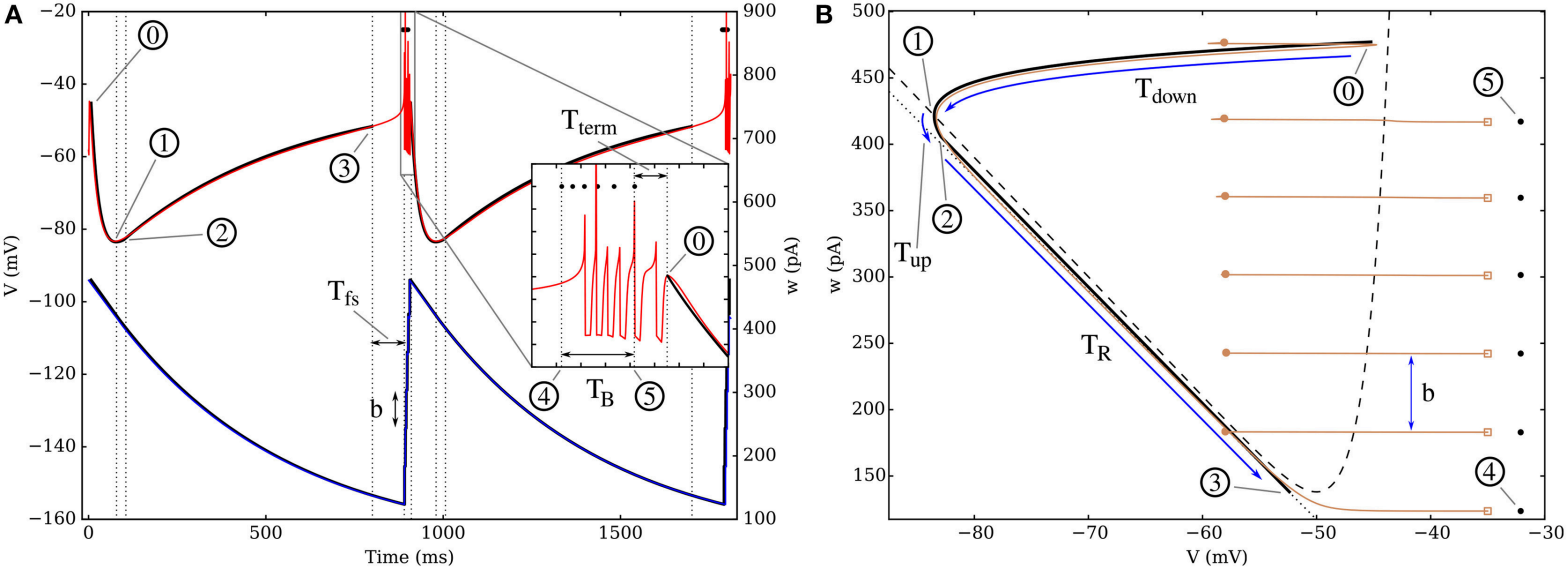
**(A)** Time evolution of parameters *V* and *w* for the theoretical model (thick black lines and circled numbers) and for a simulation (thin red curve for *V*, blue for *w*) on a fixed in-degree graph with *k* = 100. Two bursts are represented and the intraburst dynamics is presented in the inset, where the spike times predicted by the equivalent model are marked by black dots. The numbered circles mark the main points of the *theoretical* dynamics, where the behavior changes, as described in the SI. **(B)** Plot of the attractors in phase space, both for the theoretical model (thick black curve), and through a simulation (thin brown). The three first periods following a burst are denoted by blue arrows: there is first a sharp decrease of *V* down to its minimum value as it crosses the *V*-nullcline (dashes); it is followed by a short interval where the neuronal state moves rapidly toward the “recovery curve” (dotted line), which is then followed until the minimum of the *V*-nullcline and the bursting sequence. The spike trajectory is cut on the figure (marked by empty squares) and the following reset point is marked by a filled circle, as the voltage is set back to *V*_*r*_ and *w* is increased by *b*. Neuronal and synaptic parameters are detailed in Table S1, Set 1. The *w*-nullcline is outside the range of **(B)**.

From the simulation, we can record the evolution of *V* and *w* during the whole dynamics to reconstruct the trajectory of the neuronal state, both in time and in phase-space. Figure 4A represents the time evolution of the equivalent neuron (see Figure 1) during a bursting dynamics on a regular fixed in-degree network and the comparison with the trajectory predicted by the “alpha” equivalent model (see section 6 of the Supplementary Material, “A more detailed model: alpha-shaped synapses”). The close agreement between these trajectories shows that the theoretical model has a good predictive power. Indeed, the most visible discrepancy between the equivalent model and the simulations concerns the precise spike times, as shown in the inset of Figure 4A; however, though the difference can be significant on the intraburst timescale, it is in fact limited to a few milliseconds, which is negligible compared to the duration of a cycle.

The dynamics can be understood most easily when looking at *w* since its behavior can be seen as relaxation oscillations: after a burst (0), the adaptation variable undergoes a quasi-exponential decrease until it reaches its minimum value *w*_*min*_—passing through points (1) to (4). At this point, the burst starts and *w* increases rapidly toward a peak value w^*^—point (5) on Figure 4, which characterizes the trajectory, and will be derived below. Once this maximum value is reached, the neuron stops spiking, the increase of *w* stops, then the cycle starts again (see Supplementary animation online).

The evolution of *V* can then be seen as an interplay between the influence of *w*, *I*_*e*_, and the synaptic currents in the active period:

- During the burst, each new spike induces a strong depolarization of the membrane, thus leading to another spike—point (4) to (5) on the figure.
- Once *w* reaches its peak value w^*^, its influence becomes predominant and prevents the neuron from firing; once the effect of the last spike vanishes, it drives a fast hyperpolarization of the neuron down to point (1).
- After *V* has reached a quasi-equilibrium value along its nullcline, it instantaneously adapts to the slow decay of *w* and increases progressively until the trajectory reaches the lowest point of the *V*-nullcline—point (3). This recovery from the strong hyperpolarization is greatly influenced by *I*_*e*_.
- At this point, the potential starts increasing more rapidly as the first spike is initiated until the bursting starts again with (4), where the first spike predicted by the equivalent model occurs.

### Understanding the initiation and termination of a burst

**Figure 5:**
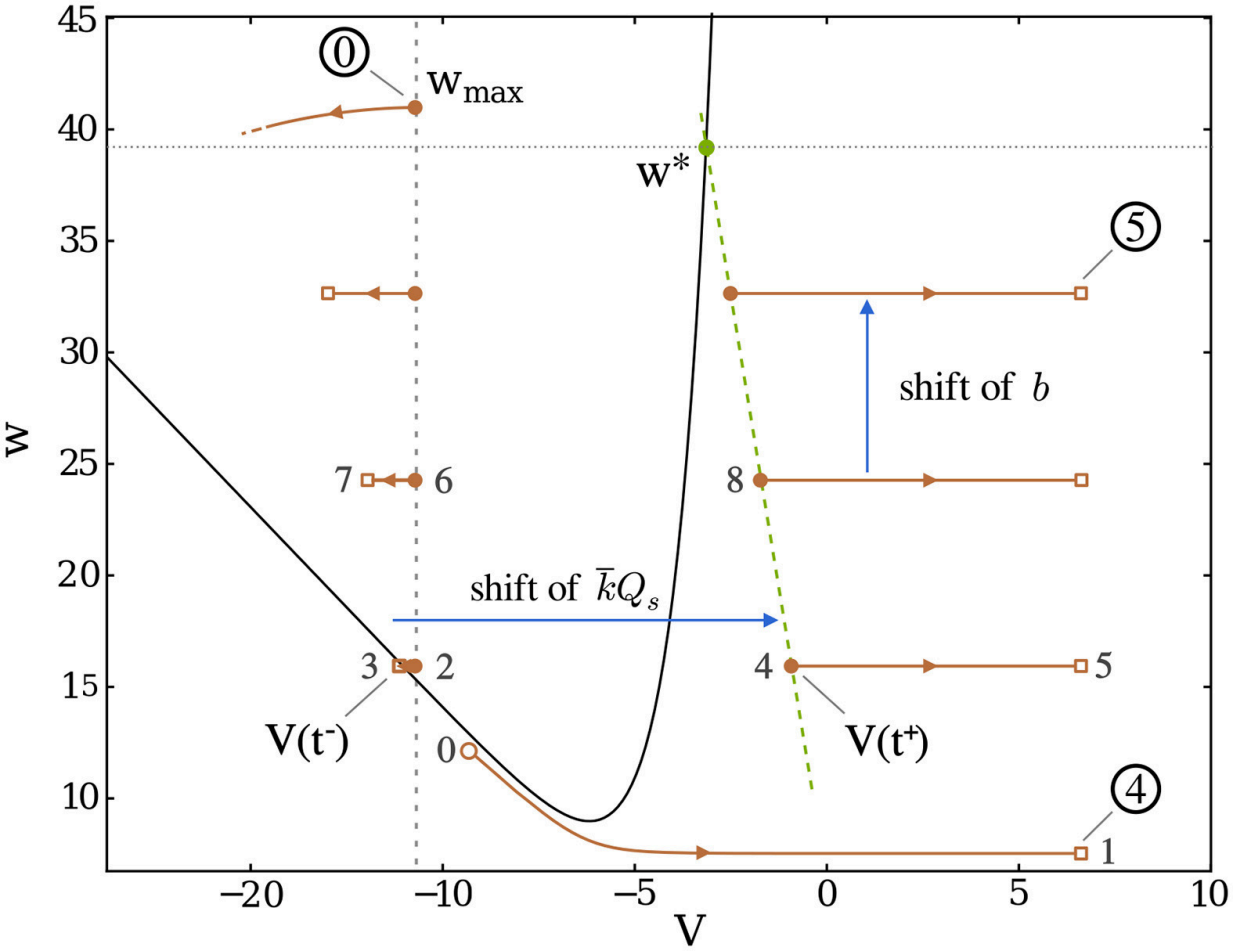
Trajectory of a “Dirac burst” in dimensionless phase space; the gray numbers indicate the order of the burst initiation. After a reset, the potential first decreases (leftmost parts of the trajectory) until the spike arrives (brown square), at which point the potential is suddenly shifted to the corresponding brown dot on the rightmost part of the trajectory. The decay before the spike arrival becomes more and more significant as *w* increases since it contributes negatively to $\dot{V}$. Burst continues until *w* becomes greater than w^*^, denoted by the green dot, where the *V*-nullcline (black line, representing the set of points (*V*_*NV*_(*w*), *w*)) is crossed. Once *w*_*max*_ is reached (circled 0), the burst ends and the recovery period starts.

One of the main interests of this equivalent model is that it provides an intuitive understanding of the mathematical conditions describing the initiation and the termination of bursts. As shown on Figures 4B, 5, the whole existence of the short active period can be understood from the position of the neuronal state in phase space compared to the *V*-nullcline (curve $\dot{V}=0$), which can be seen as an effective threshold. Indeed, the initiation of the burst simply occurs when *w* becomes low enough so that the trajectory can “pass under” the *V*-nullcline; this can be understood easily since the excitability of the neuron increases when *w* decreases. The lowest value *w*_*min*_ represent the situation where the excitability of the neuron has become so high that it spontaneously emits a spike.

A key result is then the derivation of a condition for burst termination. We show that the end of the spiking sequence that constitutes a burst is ensured by the intrinsic dynamical properties of single neurons—through adaptation mechanisms—and does not require inhibition nor plastic synapses.

To understand the succession of spikes during the burst and why this spiking process comes to an end, we must introduce a description of the dynamic coupling between the neurons. We first explicit this coupling for two limit cases: firstly instantaneous couplings in perfectly regular fixed in-degree networks, using synapses modeled by Dirac delta functions (called Dirac synapses in the following); secondly, mimicking the effect of highly disordered networks, where synapses release a constant current over the entire burst duration. Thirdly, we consider a more biologically relevant coupling using alpha-shaped synapses, detailed in section 6 of the Supplementary Material, which lies between these two previous limits.

In general, the synaptic coupling *I*_*s*_ between the neurons is time-dependent, which makes the resolution of the system's dynamics (Equation 3) highly complex. As a result the *V*-nullcline ($\dot{V}=0$) is not generally fixed over a whole cycle. This complicates the threshold condition on w^*^ in the case of the “alpha” synapses. Therefore, the Dirac and continuous synaptic models are more convenient since they enable us to get an insight on the bursting mechanisms through a static representation of the phase diagram during a burst.

#### Regular networks and dirac synapses

The rationale for the condition of burst termination is most easily understandable in the case of regular networks assuming a coupling in the form of Dirac synapses. Indeed, the arrival of a spike then simply results in a step increment of the post-synaptic neuron's membrane potential:

(9) $$V(t_{sp}^{+})=V(t_{sp}^{-})+\bar{k}Q_{s}$$

where *t*_*sp*_ is the time at which the spike is delivered to the post-synaptic neuron; $t_{sp}^{-}$, $t_{sp}^{+}$ are respectively the instants immediately before and after spike delivery. *Q*_*s*_ is the total charge delivered by the spike and reflects the coupling strength in the network.

The behavior of the neuron can easily be understood by looking at the situation in phase space on Figure 5. Due to the instantaneous coupling through the Dirac function, there is no finite period of time where the equation for *V* receives a non-zero input. Consequently, in this limit the *V*-nullcline remains fixed at all times. Therefore, the condition for the occurrence of a new spike during the burst depends only on the position of $V\left(t_{sp}^{+}\right)$ compared with the value of the *V*-nullcline at the same *w*: *V*_*NV*_(*w*). During an interspike of duration *T*_*I*_(*w*), *w* can be considered as constant since τ_*m*_, *T*_*I*_(*w*) ≪ τ_*w*_ (quasi-static approximation). Hence, either $V\left(t_{sp}^{+}\right)>V_{NV}\left(w\right)$ and a new spike occurs, or $V\left(t_{sp}^{+}\right)\leq V_{NV}\left(w\right)$ and the burst terminates.

Developing this condition mathematically leads to the following self-consistent equation:

(10) $$w^{*}=E_{L}+I_{e}-V_{r}+\left[W_{-1}\left(-e^{E_{L}+I_{e}-w^{*}}\right)+\bar{k}Q_{s}\right]e^{d}$$

where $W_{-1}$ is the lower branch of the Lambert W function.

#### Heterogeneous networks and continuous synapses

For very heterogeneous networks, the broad in-degree distribution leads the neurons to fire at seemingly random times during the bursting period. In the limit where the time distribution of the spikes inside a burst becomes completely uniform, we can approximate it through a window-like synaptic current which is zero during the interburst, then jumps to a finite constant value during the burst.

**Figure 6:**
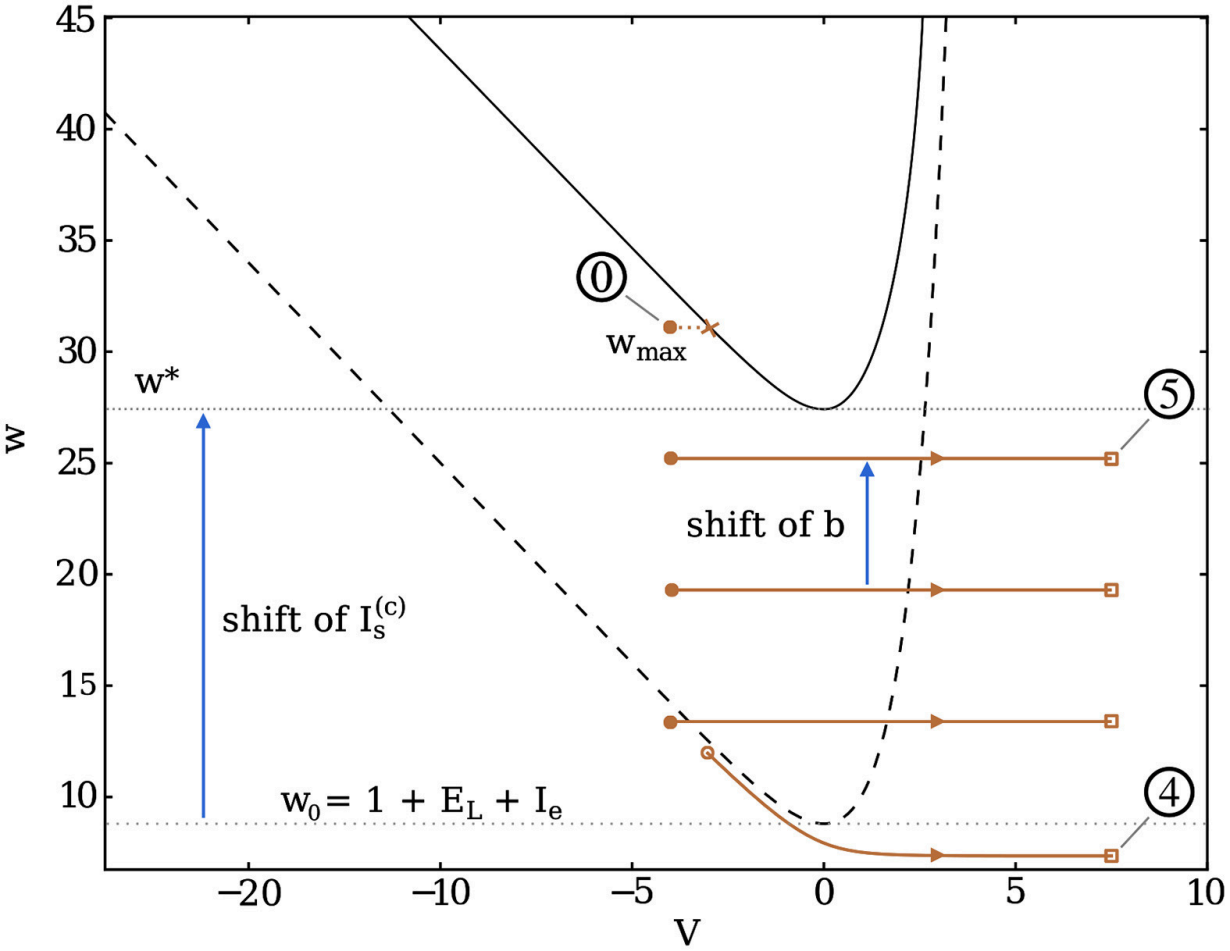
Trajectory of a burst in dimensionless phase space for neurons coupled via continuous synapses. Once the first spike occurs (marked by 4), the burst is initiated, i.e., a continuous current $I_{s}^{\left(c\right)}$ is injected into the neurons, thus shifting the resting *V*-nullcline (dashed curve) upwards (solid black). The neuron spikes until the last shift of *b* brings *w* above w^*^, at *w*_*max*_, where it encounters the nullcline. This marks the end of the burst and the beginning of the recovery period (circled 0).

To obtain an effect equivalent to the spikes described in the previous subsection, devoted the Dirac model, the total charge transmitted during the burst should be the same if an equal number of spikes is emitted. This condition reads, for an average in-degree $\bar{k}$, and a mean synaptic current $I_{s}^{\left(c\right)}$ during the burst,

(11) $$I_{s}^{\left(c\right)}T_{B}=n_{s}\bar{k}Q_{s}.$$

where *n*_*s*_ is the number of spikes inside the burst. As described previously, the burst termination occurs when the trajectory crosses the *V*-nullcline. Figure 6 shows this condition in this heterogeneous limit, i.e., as the input received by the neurons becomes continuous during the burst.

Because of the quasi-static hypothesis on *w* during an interspike, burst termination arises when *w* goes above the lowest point of the *V*-nullcline, which occurs for $w^{*}=1+E_{L}+I_{s}^{\left(c\right)}+I_{e}$. This is obtained by setting *V* = 0 in *w*_*NV*_(*V*). After a few lines of calculation detailed in the Supplementary Material (Equations S8–S10), we obtain the self-consistent equation:

(12) $$w^{*}=w_{min}+b\left[\bar{t_{s}}(w^{*})-d\right]+\bar{k}Q_{s}.$$

where $\bar{t_{s}}\left(w^{*}\right)$ is the average interspike interval (ISI) in the burst. As in the previous equations (Equations 8, 10), the critical value of the adaptation current *wj* at which the burst terminates (1) increases when the coupling strength ($\bar{k}Q_{s}$) increases (2) decreases when the transmission delay *d* increases. Furthermore, this self-consistent equation also shows the effect of the spike-driven adaptation *b* which increases the maximum value of the adaptation current that can be reached.

### Summary of the theoretical description

Once w^*^ has been computed using one of the theoretical models, we can derive all the dynamical properties, starting with:

(13) $$w_{max}=\{\begin{array}{ll} \left\lceil \frac{w^{*}-w_{min}}{b}\right\rceil b & \text{for fixed in-degree networks}\\ \;w^{*} & \text{with heterogeneity} \end{array}$$

where ⌈·⌉ denotes the ceiling function. Though the self-consistent equations derived above are less easy to interpret compared to the approximated solution (Equation 9), they allow precise quantitative predictions of the network's dynamics without too much computational cost.

Note that the neurons follow a well-defined and unique attractor, with *w* changing by discrete steps, only in the case of a fixed in-degree network, where they are all equivalent and synchronous, hence the dual form of Equation (14). In the presence of heterogeneity, the attractor has fuzzy boundaries, as shown by numerical simulations on Figure 2. In this case, the average adaptation over all neurons has a smooth dynamics and *w*_*max*_ is closer to the statistical value at which the neurons stop bursting: w^*^.

The complete dynamics of the model can be completely captured by the relaxation behavior of *w*, which displays two phases: one resting period where the adaptation variable decreases until it reaches its lowest value, and an active period where *w* increases rapidly up to its peak value. The duration of the resting period (interburst interval, or *IBI*) can be approximated as the sum of the following terms:

*T*_*down*_    characterizes the time necessary for the neuron to undergo its strong hyperpolarization and reach its lowest membrane potential—from (0) to (1) on Figure 4,
*T*_*R*_         is the duration of the recovery—from (2) to (3),
*T*_*fs*_           is the time necessary for the initiation of the first spike which is roughly equivalent to the membrane time constant τ_*m*_—from (3) to (4).

This allows us to obtain the characteristic values of the dynamics (see section 8 of the Supplementary Material, “Resting period”, for detailed calculations):

*n*_*s*_ $=\left\lceil \frac{w^{*}-w_{min}}{b}\right\rceil$ (average number of spikes in a burst),
*T*_*B*_ $=\overset{n_{s}-1}{\underset{j=1}{\sum }}t_{s}\left(w_{min}+jb\right)$, where *t*_*s*_(*w*) is the interspike interval (ISI) for a given value of *w*,
*T*_*down*_ $=\ln\left(\frac{\lambda }{\lambda -V_{max}+E_{L}+I_{e}-w_{max}}\right)$,
*T*_*R*_ $=\frac{\tau_{w}-a}{1+a}\ln\left(\frac{w^{\left(2\right)}-\frac{a}{1+a}I_{e}}{w_{min}-\frac{a}{1+a}I_{e}}\right)$, where *w*^(2)^ is the value of *w* at point (2),
*IBI* ≈ *T*_*down*_ + *T*_*R*_ + 1.

Because these results are analytic, thus immediate to compute, this has the significant advantage over simulations that it allows us to quickly predict the properties of the collective dynamics for a large number of parameter sets, i.e., of individual neuron's behaviors.

### Evolution of the properties with neuronal and synaptic parameters

**Figure 7:**
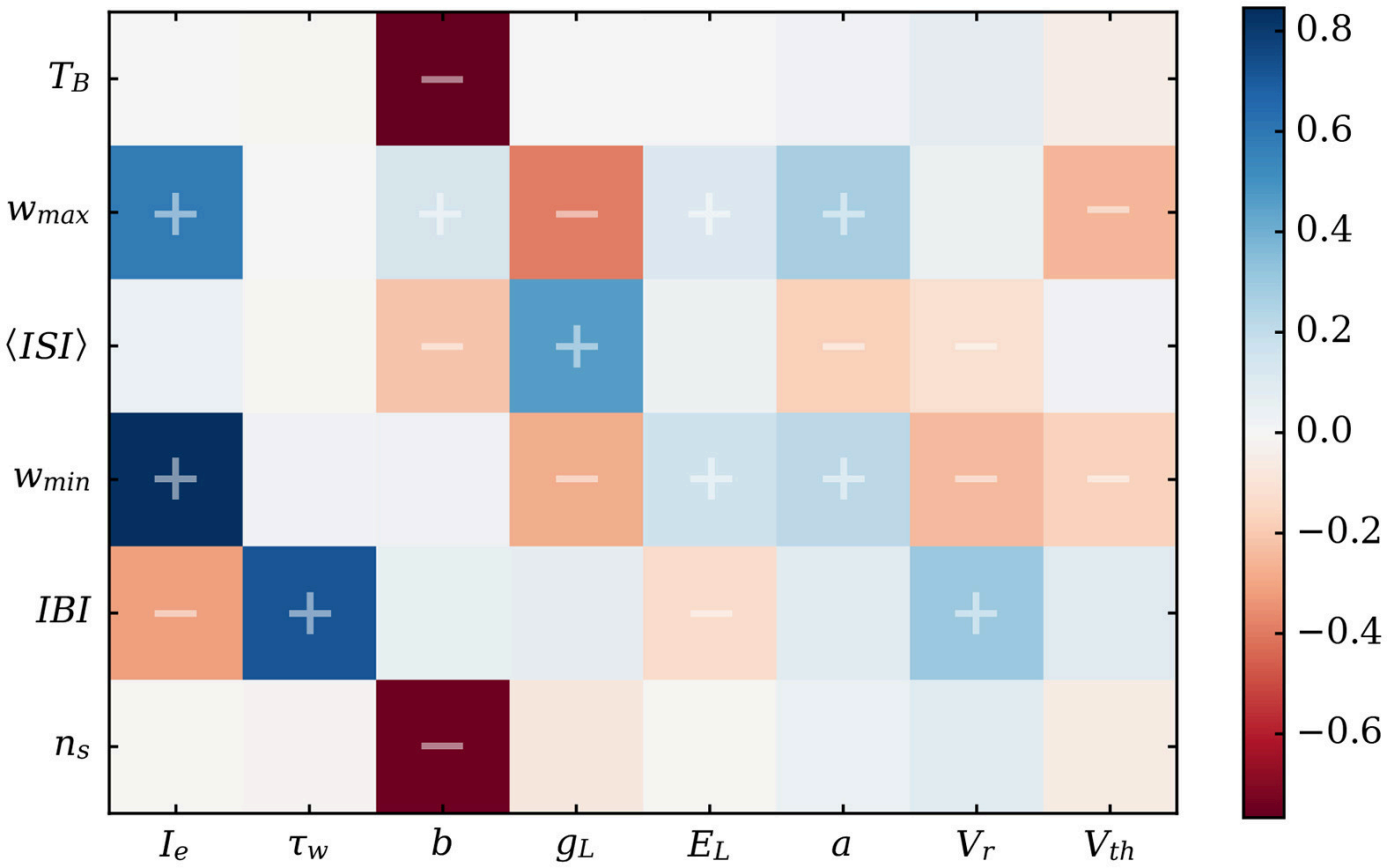
Correlation matrix for the main characteristics of the bursting dynamics vs. neuronal parameters. 〈*ISI*〉 is the mean value of the interspike over one burst. Correlations were performed over 2 million randomly-drawn neuronal parameter sets using the predictions of the equivalent model. The experimentally observable features are the *IBI*, *T*_*B*_, *n*_*s*_, and 〈*ISI*〉.

In order to assess the separate influence of the different neuronal parameters on the bursting properties, we used the model to test in a systematic way the influence of the separate variables. As can be seen on Figure 7, this allows to compare the relative influence of any desired set of parameters in a fast and systematic way. Thus, it is a valuable tool to make preliminary explorations in order to prepare for subsequent experimental tests.

This matrix allows us to confirm obvious trends, such as the negative influence of the driving current *I*_*e*_ on the *IBI*, as it tends to quicken the depolarization of the neurons. Likewise, τ_*w*_ is almost linearly related to the *IBI* since it dictates the decay time for *w*. However, this systematic study also revealed less predictable correlations. Indeed, one of the most interesting features is the quasi-absence of influence of the subthreshold adaptation variable *a* compared to the spike-driven adaptation (characterized by *b* and *V*_*r*_) on the most visible features of the activity, namely the *IBI* and burst duration.

Correlations for 〈*ISI*〉 should be treated with care as this value is the average of the interspike interval over a burst. An additional spike (increment in *n*_*s*_) automatically increases 〈*ISI*〉 since the new interspike interval will be larger than the previous ones. This is due to the monotonic growth of *ISI* with *w* as the burst progresses. Thus, if *V*_*r*_ indeed reduces the interspike duration on the whole, the negative correlation between 〈*ISI*〉 and *b* mainly comes from the decrease of *n*_*s*_ as *b* increases.

**Figure 8:**
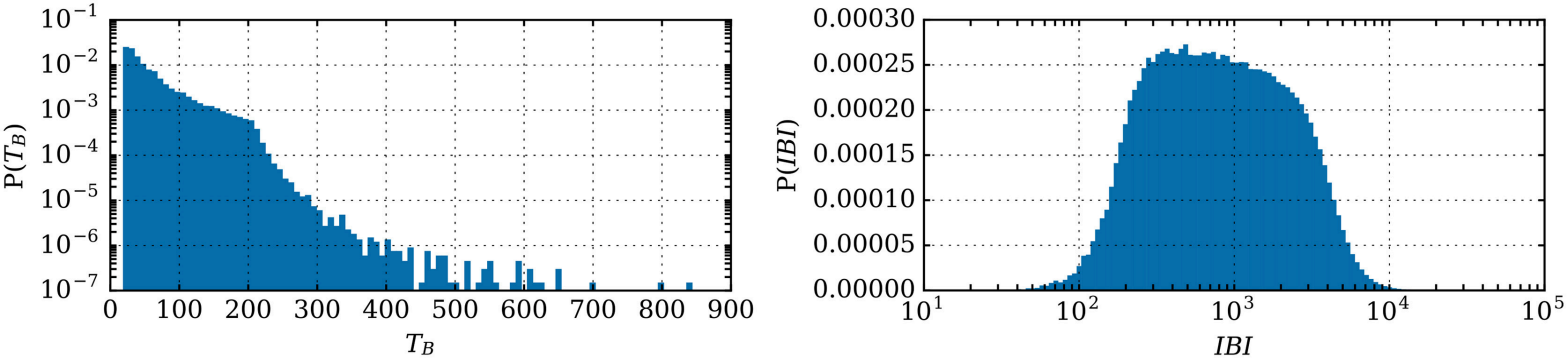
Distributions of the burst burst duration *T*_*B*_ and of the *IBI* (both in ms) for 2 million parameter sets.

Due to the sheer amount of calculation this would require, the theoretical values returned by the equivalent model during this large exploration of parameter-space cannot be verified by simulations in a systematic way. However, the distributions of the bursting characteristics (number of spikes, interburst, and burst duration) are in biologically relevant ranges—see Figure 8. This shows that adaptation alone can lead to network bursts with periods varying from a few tenths of milliseconds to several seconds.

### Predictive ability of the burst model for heterogeneous networks

**Figure 9:**
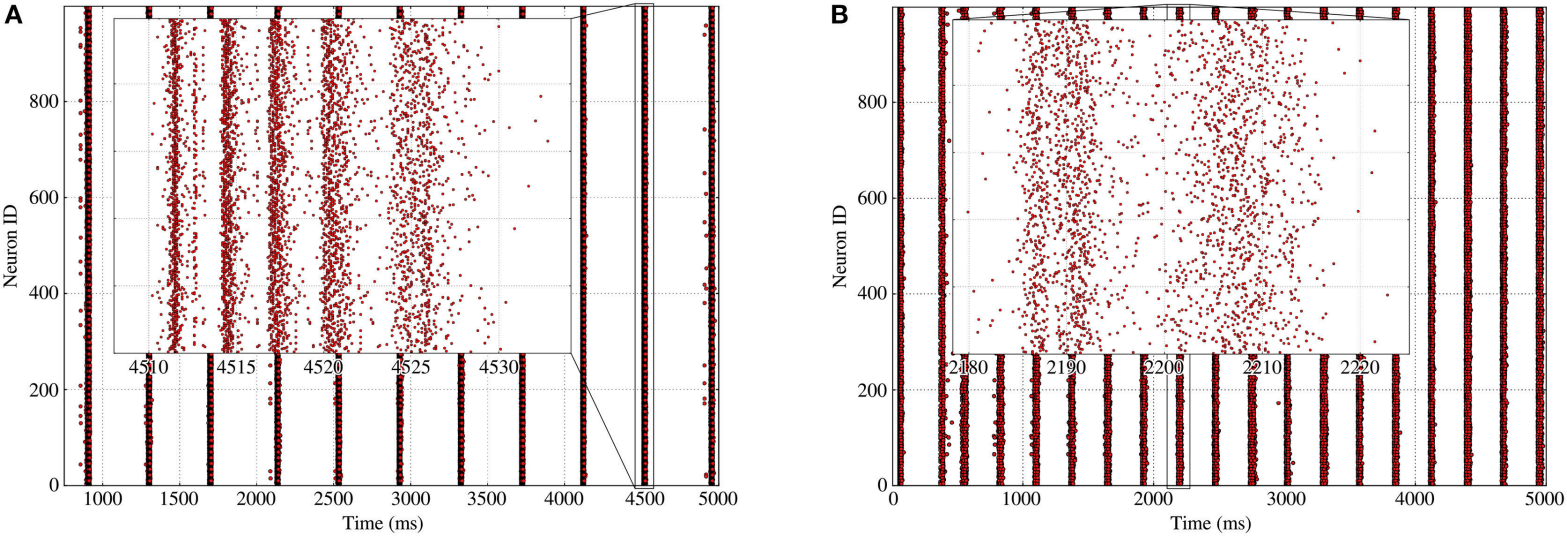
Rasters of the bursting activity for 2 different Gaussian networks with 1,000 neurons and an average in-degree of 100; each inset details the inner structure of a burst with the successive slices. **(A)** Homogeneous Gaussian in-degree network with $\bar{k}=100$ and σ_*k*_ = 5 leads to well-defined synchronized burst slices inside the bursts. **(B)** Heterogeneous Gaussian in-degree network with $\bar{k}=100$ and σ_*k*_ = 20 leads to fuzzy synchronized burst slices.

Our description of periodic bursts predicts the main features of the synchronized bursting rhythmic activity such as its period and firing rate, which are significantly influenced by the presence of heterogeneity in the network's structure, as was already visible in Figure 2. Indeed, as the heterogeneity—namely σ_*k*_—increases, the sharpness of the synchronized burst slices decreases until the spikes contained in the burst become more uniformly distributed; this is clearly visible on Figure 9, which shows the comparison of a burst for two Gaussian in-degree networks with different standard deviations.

**Figure 10:**
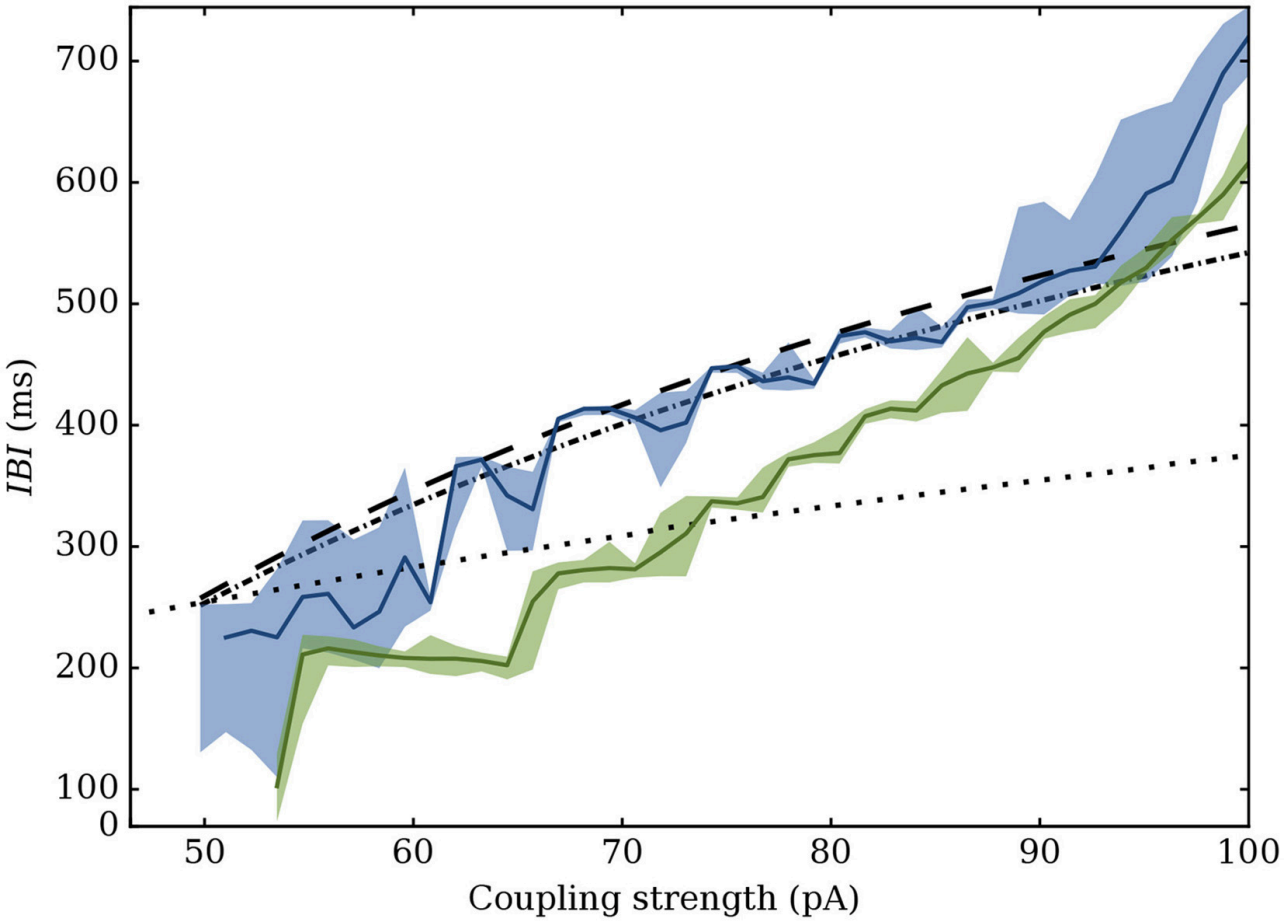
Variation of the *IBI* depending on maximum value of the PSC (in pA). Values predicted by the equivalent model are shown in dashed, dot-dashed and dotted lines respectively for the Dirac, alpha and continuous models. Simulated values for a Gaussian network with σ_*k*_ = 4 (blue) and σ_*k*_ = 20 (green) are superimposed: the main curve represents the average value, while the filled area marks the 5th to 95th percentiles.

Our model is able to take this heterogeneity into account through three synaptic descriptions (Dirac, alpha-shaped, or “continuous”): this allows us to predict the interval in which the bursting properties of most networks should be contained. As shown on Figure 10, they fall in between the Dirac and “continuous-synapses” models. This description successfully accounts for dynamics of networks with low heterogeneity. For high levels of heterogeneity and low synaptic strengths, the model tends to overestimate the synchrony, although prediction of the bursting period remains correct.

## Discussion

In all the simulations we performed, we observed that oscillating adaptive spiking neurons synchronize, then start emitting bursts of spikes as the coupling increases.

Our model provides a predictive framework which allows us to determine how this bursting behavior is affected by changes in the individual properties of the neurons.

In the following subsections, we first discuss the validity range of the analytic model. Then, through a thorough mapping of the aEIF parameters to ion channels and biological mechanisms, we make experimentally-testable predictions about the possible influence the main adaptation channels on the bursting behavior. Namely, we suggest how adaptation-channel blockers may affect the dynamics when applied on a bursting neuronal culture.

### Validity range of the equivalent model

In order to get meaningful results within the framework of the present model, one must take care to use sets of parameters that lead to adaptive spiking neurons.

More importantly, the conceptual boundaries of the model are reached in the limit of either a very weakly or very strongly coupled neuronal network. For strong coupling the discrepancy between the equivalent model and the simulations mostly occurs because PSCs becomes so intense that a single input can generate several spikes. This can occur *in silico* but has little biological relevance for adaptive spiking neurons. The weak coupling limit, however, is more revealing since a progressive transition from an asynchronous state to a bursting phase occurs. This transition first involves oscillating firing rates, then synchronous slices containing between one and two spikes, before bursts containing multiple spikes appear. Our equivalent model, designed to describe a fully synchronous bursting dynamics, cannot faithfully capture this smooth transition.

Regarding the network structure, more heterogeneous (e.g., scale-free) networks may also be described by the “continuous-synapse” model on some range of the coupling strength as the qualitative bursting behavior is still present on such networks.

### The influence of adaptation and its biological origin

Despite its simplicity, the aEIF model takes into account most of the adaptation phenomena involved in biological neurons. Thus, voltage-gated subthreshold adaptation currents, like the muscarinic potassium current *I*_*M*_ (Womble and Moises, 1992) are quantified by the constant *a* in Equation (3). On the other hand, spike-triggered adaptation, which mostly comes from calcium-gated potassium channels leading to after-hyperpolarization (AHP) phenomena (Sah and Louise Faber, 2002), are quantified by the reset conditions. These calcium-activated currents can be separated into three main types (Sah, 1996; Sah and Louise Faber, 2002; Vogalis et al., 2003) according to their timescales. Over a few milliseconds (1–10 ms) the fast hyperpolarization current *fAHP* contributes to action potential repolarization, and is thus taken into account by the model through the value of *V*_*r*_ in Equation (1). On an intermediate (“medium”) timescale, the current *mAHP* has a fast rise-time (less than 10 ms), followed by a decay over 50 to several hundred milliseconds (Storm, 1989); it is modeled by the *b* step of *w* after a spike, in Equation (2). Finally, the slow hyperpolarization (Shah and Haylett, 2000; Andrade et al., 2012) current *sAHP* has a slow rise of 100 ms or more, and an even slower decay over several seconds. It is mostly revealed after a train of action potentials and peaks between 400 and 700 ms. Though this current is not explicitly taken into account by the aEIF model, in the case of bursts, its qualitative effect can be obtained approximately by an increase of τ_*w*_, which lengthens the effect of the potassium current after a burst. One of the limits of the model is its unique timescale for all of the adaptation-related features.

From the exploration of parameter-space, we obtain the correlation matrix of Figure 7, which shows a significant influence of spike-triggered adaptation on the dynamics compared to subthreshold adaptation. A previous study (Augustin et al., 2013) also hinted at the importance of a non-zero *b* value to obtain low-frequency oscillations. Using the equivalent model, this can be explained easily by the quasi-static hypothesis and the shape of Equation (13). Indeed, the second term of the right-hand side involves the average ISI—which is an increasing function of w^*^—and the spike-driven increment for the adaptation, *b*. Thus, the higher the effect of the spike-driven adaptation, the higher w^*^, which leads to longer interbursts. On the other hand, the quasi-static hypothesis states that the evolution of *w* is slow compared to that of *V*, meaning that the subthreshold variations given by *a* are limited by their slow evolution on a timescale of τ_*w*_.

A significant advantage of this simple description is that the mechanisms proposed by our equivalent model, in light of the correlation matrix on Figure 7, allow us to make qualitative predictions that could be tested to validate it experimentally. Thus, we predict that blocking the voltage-gated adaptation (Stiefel et al., 2008) should have only limited influence on the dynamics through a slight increase in the number of spikes during a burst. On the contrary, blocking one of the calcium-gated channels should lead to drastic changes in the collective behavior:

- Blocking the *fAHP* channels should be equivalent to increasing *V*_*r*_, hence increasing the number of spikes in a burst, leading to higher *w*_*max*_, therefore longer *IBI*.
- Blocking the *mAHP* channels through apamin (Sah and Louise Faber, 2002) would be equivalent to lowering the value of *b*, which should strongly impact the number of spikes inside a burst, therefore its duration. Yet, this should not change *w*_*max*_ significantly, so it should not strongly impact the *IBI* if the *sAHP* is significant enough. However, in the case of complete blocking, if the *sAHP* is not strong enough to compensate, this should lead to the complete disappearance of the bursting behavior.
- Specific blocking of *sAHP* channels via noradrenaline (Sah and Louise Faber, 2002) should lead to a small increase of the number of spikes during a burst, but would mostly be equivalent to lowering τ_*w*_. In situation where adaptation has the strongest influence over the bursting period, this would lead to a significant decrease of the *IBI*. This is however unlikely to happen in neuronal cultures, as will be explained below.

These experiments would enable to test the adaptation hypothesis and assess the relative strength of the different processes we described. In fact, some previous studies by Empson and Jefferys (2001) and de Sevilla et al. (2006) have shown results that seem to corroborate the previous predictions, at least regarding the effect of apamin on bursting in slices. However, the first study records only from few individual neurons, and the second uses 4-aminopyridine and Mg^2+^-free medium to trigger the epileptiform activity. To assess the general validity of the proposed mechanisms, one would thus need additional measurements using cultures in physiological conditions, and where each ion-channel would be tested independently while recording larger fractions of the network, either through calcium imaging or MEAs.

**Figure 11:**
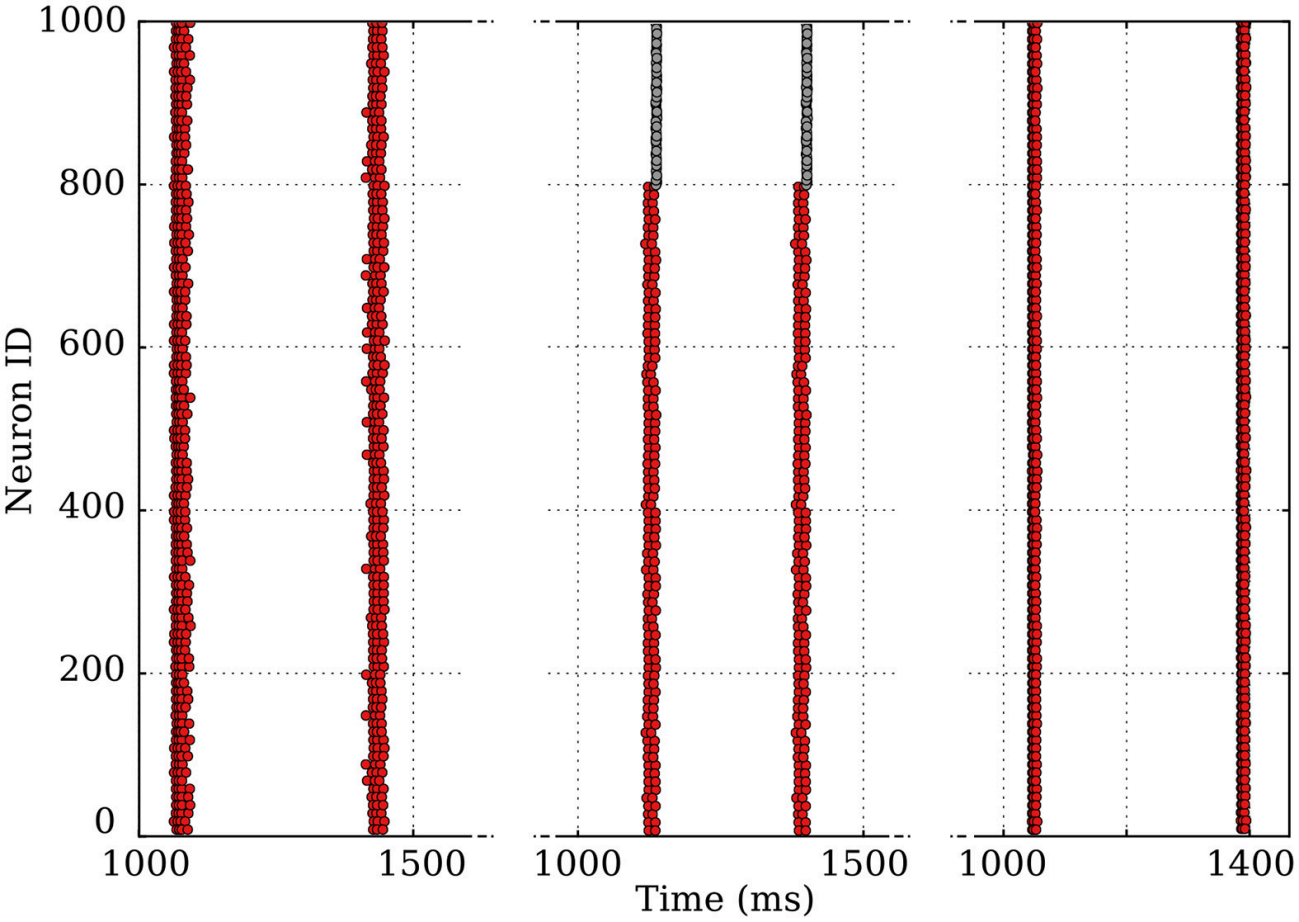
Modification of the original dynamics (left), where only excitatory neurons are present, by the introduction of 20% of non-oscillating, fast-spiking inhibitory neurons (middle), or of plastic synapses exhibiting short-term depression (right). The coherence of the qualitative aspect over three very different systems is remarkable.

Moreover, other features, such as slow modulation of extracellular potassium concentration due to neuronal activity (Bazhenov et al., 2008) have been described in the context of rhythmic activities; these experiments would also help determine whether such phenomena are required as driving forces or only contribute to strengthening existing bursting activities. In our simulations, network bursting is very robust against the following modifications of the system: addition of inhibitory neurons in the network or inclusion of short-time depression in the synaptic dynamics, as shown on Figure 11. It is thus likely that the underlying mechanism we detailed for excitatory synapses can be generalized to these cases. Indeed, the mechanism remains unchanged by plasticity, while adding inhibitory neurons in the population essentially translates into an effective decrease of the excitatory coupling; the latter has been pointed out for percolation in networks of integrate-and-fire neurons.

Eventually, previous studies (Cohen and Segal, 2011) have hinted at the importance of synaptic fatigue in the burst termination: they showed that the duration of an evoked burst was strongly dependent on the elapsed time since the previous burst, due to the time needed to repopulate the pools of neurotransmitters. What we showed here is also compatible with these results, since they can easily be understood in the framework of our model: a smaller recovery time leads to a higher initial value of the adaptation current, thus shortening the burst duration because the maximum value of *w* is reached sooner. The effects of synaptic plasticity and adaptation should thus be similar; however, given the timescales reported in the literature, termination could be mostly mediated by adaptation, while the *IBI* might depend more strongly on synaptic recovery time. In such a case, blocking sAHP as proposed above should not dramatically change the *IBI* of neuronal cultures.

## Conclusion

This study explains the dynamical processes determining synchronous network bursting of a population of oscillating neurons coupled through excitatory synapses. In particular we explain why adaptation is a sufficient condition for collective bursting. We reproduce a large range of biological rhythms with burst frequencies spanning almost 3 orders of magnitude, from a few hundred milliseconds to tens of seconds, in agreement with experimental observations.

Thanks to a phase-space analysis, we are able to propose a mechanism for the initiation and termination of the bursting period related to spike-driven adaptation, which we link to the underlying biological phenomena. The derivation of analytic equivalent models describing the complete bursting dynamics allows us to predict the evolution of the characteristics of the global behavior from the properties of the individual units—neurons and synapses. This enables us to propose a set of experiments which should clarify the role of adaptation currents in network bursting, as well as their relative importance compared to other biological processes such as exhaustion of vesicle pools.

In our description, each new spike in the burst is caused by the previous one, which means that the delay between the emission of a spike and its reception by the post-synaptic neuron has a significant influence on the dynamics. Indeed, we understand intuitively that the longer the delay, the lower the excitability of the neurons when the PSC arrives, since the membrane potential can decay to lower values. This fact, added to the effect of heterogeneity—which tends to reduce the interburst interval—hints at the existence of a limit to the spatial extension which can sustain coherent bursting. Exploring the effect of heterogeneity and spatial embedding (through propagation delays) therefore constitutes a natural continuation of this work. This is certainly necessary to address experimental observations in large cultures, such as the tendency of the activity to initiate in specific regions before it propagates to the rest of the network (Orlandi et al., 2013).

## Author contributions

Analytical study was conducted by MB and TF. TF also performed the numerical simulations and redaction of first draft. Additional simulations and analytical work were performed, respectively, by PM and SM. Final restructuring and adjustments were performed together by SB, TF, SM, and PM.

### Conflict of interest statement

The authors declare that the research was conducted in the absence of any commercial or financial relationships that could be construed as a potential conflict of interest.

## Footnotes

^1^Let us insist once again that the term *burst* always refers here to the concerted activity of a large fraction of the neuronal population (i.e., a *network burst*) and should not be confused with single neuron bursting behavior—though they share common characteristics—since they have different origins and population bursts occur on much longer timescales.

## Supplementary material

All codes used in this article are available on our GitHub repositories (https://github.com/SENeC-Initiative/PyNeurActiv and https://github.com/SENeC-Initiative/figures_examples). Note that some of the code might require additional libraries such as NNGT or NEST to run.
