# Supplementary material for: Understanding the Generation of Network Bursts by Adaptive Oscillatory Neurons
Source: Front Neurosci. 2018 Feb 6;12:41. doi: 10.3389/fnins.2018.00041 (PMC5808224; doi:10.3389/fnins.2018.00041)
Supplement: Supplementary file 2 [file DataSheet1.PDF]

Supplementary Material: Adaptive oscillatory neurons are sufficient to generate network bursts

1 NEURONAL MODEL (ADEX) AND PARAMETERS

1.1 Dimensionless parameters

The dimensionless parameters are obtained from their dimensional counterparts via the following formulas:

$$V = \frac{\tilde{V} - \tilde{V}_{th}}{\tilde{\Delta}_T}, \qquad E_L = \frac{\tilde{E}_L - \tilde{V}_{th}}{\tilde{\Delta}_T} \qquad \text{(general relation for voltages)}$$

$$w = \frac{\tilde{w}}{\tilde{g}_L \tilde{\Delta}_T}, \qquad I = \frac{\tilde{I}}{\tilde{g}_L \tilde{\Delta}_T} \qquad \text{(general relation for currents)}$$

$$t = \frac{\tilde{t}}{\tilde{\tau}_m}, \qquad \tau_w = \frac{\tilde{\tau}_w}{\tilde{\tau}_m} \qquad \text{(general relation for times)}$$

$$g_L = 1 \qquad a = \frac{\tilde{a}}{\tilde{g}_L} \qquad \text{(general relation for conductances)}$$

1.2 Parameter sets and relevant range

Throughout the numerical simulations, we restricted ourselves to a biologically relevant range for each parameters. This range, as well as two specific sets of parameters are detailed in Table S1.

Parameter	Range	Set 1	Set 2
$\tilde{C}_m$	200	200	200
$\tilde{g}_L$	[4; 15]	9	9
$\tilde{E}_L$	$[-75; -65]$	-70	-70
$\tilde{V}_{th}$	$[-60; -45]$	-50	-50
$\tilde{V}_r$	$[-70; -50]$	-58	-58
$\tilde{I}_e$	[300; 600]	300	300
$\tilde{\Delta}_T$	2	2	2
$\tilde{a}$	[0; 15]	2	2
$\tilde{b}$	[2; 150]	60	5
$\tilde{V}_{peak}$	0	0	0
$\tilde{\tau}_w$	[300; 12000]	300	2000
$\tilde{t}_{ref}$	0	0	0
$\tilde{\tau}_s$	[0.2; 0.8]	0.2	0.7
$\tilde{d}$	[1; 30]	1	5

Table S1. Neuronal and synaptic parameters used in the simulations. The units are as follow: capacitance in pF, conductance in nS, voltage in mV, current in pA and time in ms. $\tilde{d}$ is the spike transmission delay and $\tilde{t}_{ref}$ is the value of the refractory period that can be set in the NEST simulator.

Though the ranges of the parameters are all biologically relevant, some combinations are not; thus, during the phase-space exploration we performed, we ruled out the sets that led to either stable equilibria for the neurons, non-real results in the self-consistent equation, or non-plausible values for one of the dynamical characteristics. This led to the following correlation matrix for the parameters.

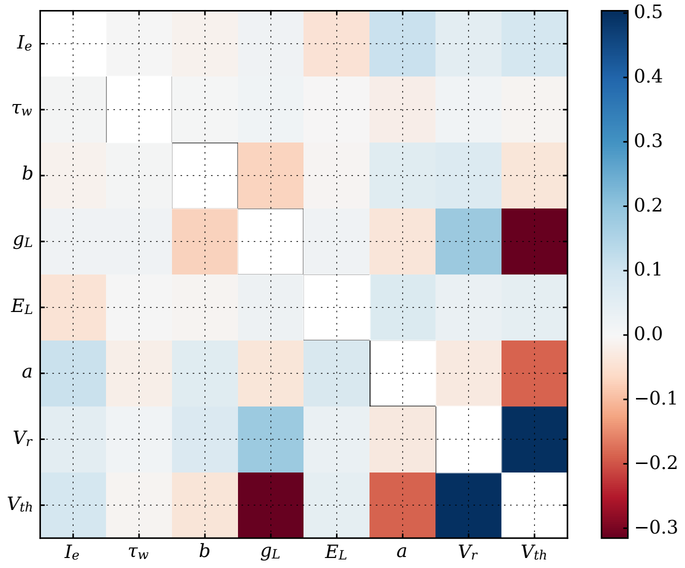

Figure S1: Correlation matrix for the parameters (2 million randomly-drawn neuronal parameters sets) after the tests for non-real or non-biological values were performed.

1.3 Adaptive oscillatory neurons and intrinsically bursting neurons

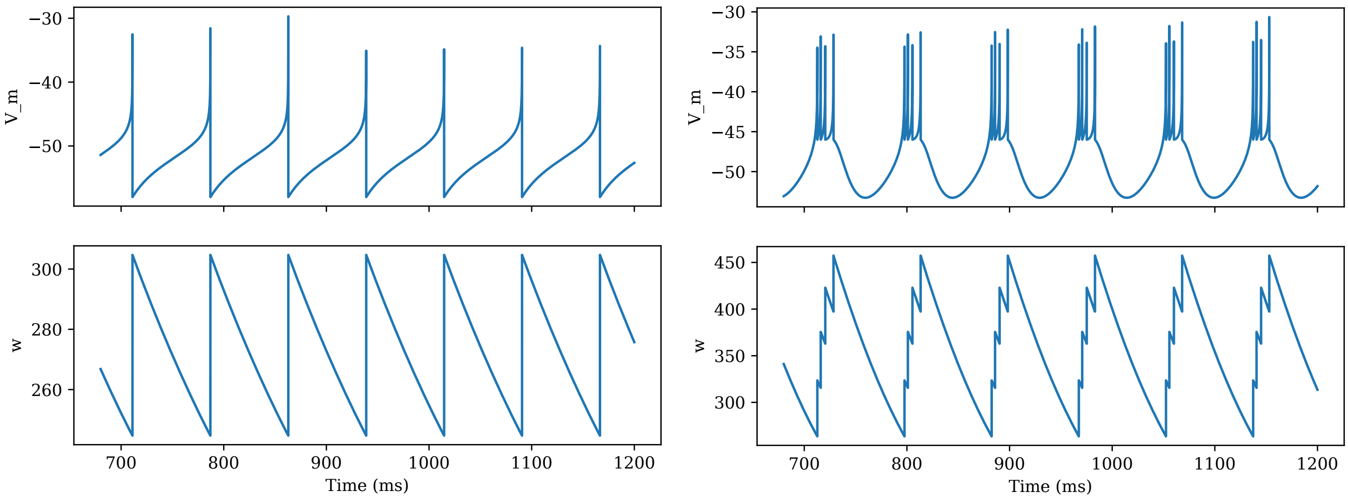

Figure S2: Time evolution of the membrane potential (top) and adaptation current (bottom) for adaptive oscillatory neurons (left) and intrinsically bursting neurons (right) modeled by the AdExp system in their permanent regimes. The neurons receive no external stimuli. The behavior of the adaptive oscillatory neurons is similar to the response of tonic spiking neurons to a step current and their peak firing rate is well below that of the intrinsically bursting neurons, which goes above 150 Hz during the bursts.

In order to show the significant difference between adaptive oscillatory neurons and intrinsically bursting neurons, Figures S2 and S3 show the trajectories of uncoupled neurons in time and phase space for the two different behaviors. Using the AdExp model, the two states differ mostly through the value of their reset potential $\tilde{V}_r$ (see S2). Indeed, bursting behavior arises for $V_r > V_{th}$, while spiking usually requires $V_r < V_{th}$.

The difference between the two behaviors is quite visible when they are uncoupled. However, since the adaptive oscillatory neurons switch to a collective bursting behavior when strongly coupled, what is really interesting is to compare the phase-space trajectory of the bursting state (e.g. Figure 4B in the main text) with the trajectory of an intrinsically bursting neuron (Figure S3, right).
Though the bursting trajectories might look similar, it is important to note that, in the case of intrinsically bursting neurons (Figure S3, right), the V-nullcline is fixed and the trajectory during the active part of the burst (where spikes are elicited) remains on the right-side of the V-nullcline and does not cross it. On the contrary, for network bursts (Figure 4B), the V-nullcline moves up and down because of incoming PSCs (see supplementary video) and the trajectory of the neural state is not confined to the right of the V-nullcline but enters its convex region after each spike-triggered reset; it is only after the arrival of the spikes from the other neurons that it can escape from inside the V-nullcline and emit a new spike.

2 RESILIENCE OF THE SYNCHRONIZATION PHENOMENON

We verified numerically that bursting synchronization always appears above a certain coupling strength $Q_{s,c}$, which precise value depends on neuronal parameters and network topology. In the range of network sizes considered – between 1000 and 100,000 neurons – the neurons rapidly switch from their initially

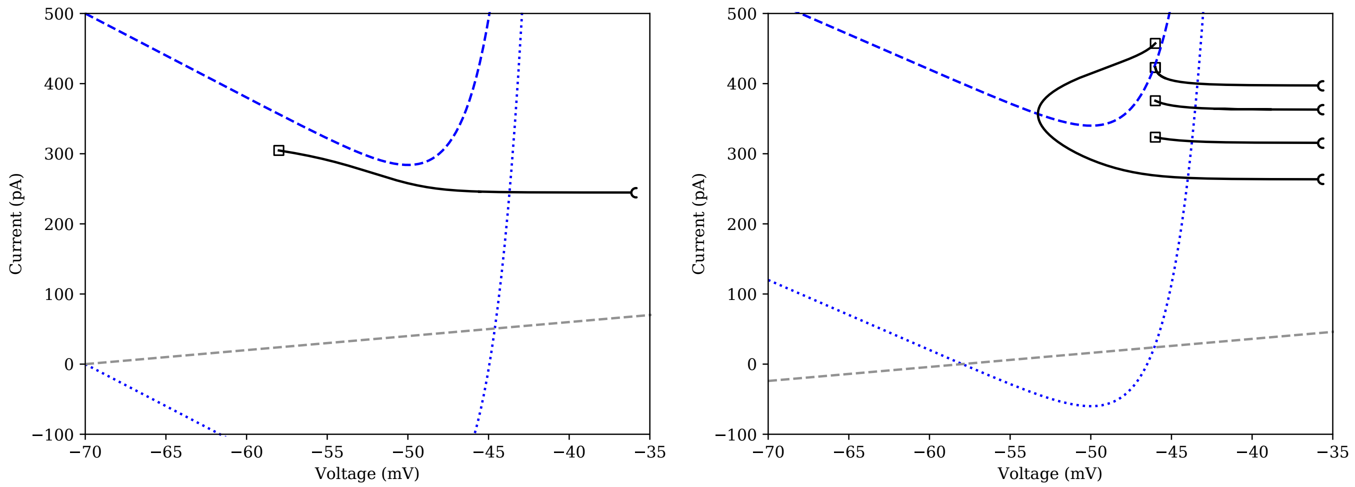

Figure S3: Trajectory of the neuronal state in phase space for adaptive oscillatory neurons (left) and intrinsically bursting neurons (right) modeled by the AdExp system and in their permanent regimes. The neurons receive no external stimuli. The cutoff after each spike is marked by an exclusive half-circle and the subsequent reset points are denoted by empty squares. The y-axis represents the adaptation current w and the x-axis is the membrane potential. The w-nullcline is the grey dashed line, the resting V-nullcline is represented by the dotted blue curve, and the intersection with the w-nullcline is the resting state, i.e. when $I_e = 0$. In the permanent oscillatory regime ($I_e > 0$), the V-nullcline is the blue dashed curve which is moved upwards compared to the resting one due to the increased current $\tilde{I}_e$ to which the neurons are subjected to put them in an active state.

Parameter	Adaptive	Bursting
$\tilde{C}_m$	200	200
$\tilde{g}_L$	12	10
$\tilde{E}_L$	-70	-58
$\tilde{V}_{th}$	-50	-50
V_r	-58	-46
$\tilde{I}_e$	500	300
Δ_T	2	2
$\tilde{a}$	2	2
$\tilde{b}$	60	60
$\tilde{\tau}_w$	300	120
$\tilde{t}_{ref}$	0	0

Table S2. Neuronal parameters used to compare the adaptive and bursting oscillatory regimes.

asynchronous state to a synchronous bursting phase above this critical value. This synchronization can be understood through an analogy with the behavior of relaxation oscillators because of the simple evolution of the slow variable w (see Figure 4B in the main text). Relaxation oscillators are known to synchronize when coupled under a broad set of conditions Somers and Kopell (1993); Bottani (1995); Izhikevich (2000); Fox et al. (2001).

3 HYPOTHESES UNDERLYING THE EQUIVALENT MODEL

As the dynamics of w is slow except for its fast spike-triggered increase during the burst, our model is based on a quasi-static hypothesis regarding the subthreshold dynamics of w:

$$\tau_w \gg \tau_m = \frac{C_m}{g_L} = 1 \tag{S1}$$

4 LINEAR EVOLUTION OF V SUBMITTED TO A CONSTANT CURRENT

During an ISI, V undergoes a quasi-linear variation which starts right after the reset following a spike (let us simplify the calculations by setting $t = 0$ at this time) and lasts until the effect of the exponential term is no longer negligible.

During this period, the membrane potential behaves as if it were submitted to the linear equation:

$$\frac{dV_l}{dt} = -(V_l - E_L) + I(t) - w \tag{S2}$$

For time independent synaptic currents, $I(t) = I$, we obtain – with $V_l(0) = V_r$:

$$V_l(t) = V_r e^{-t} + (E_L + I - w)\left(1 - e^{-t}\right) \tag{S3}$$

5 MODELING A BURST WITH INSTANTANEOUS DIRAC SYNAPSES

In all this section, the current to which the neurons are subjected is $I(t) = I_0$ at all times and the effect of the spike is instantaneously delivered as a Dirac pulse, i.e. as an equivalent charge Q_s.

5.1 Interspike approximation

Between two spikes the slow current w varies slowly enough to be taken constant – see Figure 6 in the main text; therefore the interspike for a given w is called $t_s(w)$. Consider $\tilde{a}$ and $\tilde{b}$ two such spikes, characterized by the times t_A and t_B, the interspike is defined as

$$t_s^{(d)}(w) = t_B - t_A = \int_{t_A}^{t_B} dt.$$

The synaptic delay after which the PSP associated to spike $\tilde{a}$ occurs separates the equation into:

$$t_s^{(d)}(w) = \int_{t_A}^{t_A+d} dt + \int_{t_A+d}^{t_B} dt.$$

Changing variables from t to V for the second term and defining $V_d^+ = V(d^+) \approx V_l(d) + \overline{k}Q_s$ from Eq. (S2), this becomes:

$$\begin{aligned} t_s^{(c)}(w) &= \int_0^d dt + \int_{V_d^+}^{V_p} \frac{dV}{e^V + E_L + I_e - w} \\ &\approx d + T_{div}^{(d)} \end{aligned}$$

where $V_d = V_l(d)$, from Eq. (S2), $\alpha = E_L + I_e - w$, $\beta = e^{V_p} + \alpha/2$, $\gamma = \exp(V_d + \overline{k}Q_s) + \alpha/2$, and

$$\begin{aligned} T_{div}^{(d)}(w) &= \int_{e^{V_d + \overline{k}Q_s}}^{e^{V_p}} \frac{du}{(u + \alpha/2)^2 - \alpha^2/4} \\ &= \int_{\gamma}^{\beta} \frac{dx}{x^2 - \alpha^2/4} \\ &= \frac{1}{\alpha} \int_{\gamma}^{\beta} \frac{dx}{x - \alpha/2} - \frac{dx}{x + \alpha/2} \\ &= \frac{1}{\alpha} \ln \left| \frac{(\gamma + \alpha/2)(\beta - \alpha/2)}{(\gamma - \alpha/2)(\beta + \alpha/2)} \right|. \end{aligned}$$

Hence:

$$t_s^{(d)}(w) \approx d + \frac{1}{\alpha} \ln \left| \frac{(2\gamma + \alpha)(2\beta - \alpha)}{(2\gamma - \alpha)(2\beta + \alpha)} \right| \qquad \text{(S4)}$$

5.2 Self-consistent equation

Using the linear solution of Eq. S2 with $I = I_e$, the condition $V(t_{sp}^+) \leq V_{NV}(w)$ translates into:

$$V_r e^{-d} + (E_L + I_e - w)(1 - e^{-d}) + \overline{k}Q_s = V_{NV}(w) \qquad \text{(S5)}$$

hence, with $V_{NV} = E_L + I_e - w - \mathcal{W}_{-1}\left(-e^{E_L + I_e - w}\right)$,

$$w^* = E_L + I_e - V_r + \left[\mathcal{W}_{-1}\left(-e^{E_L + I_e - w^*}\right) + \overline{k}Q_s\right] e^d \qquad \text{(S6)}$$

where $\mathcal{W}_{-1}$ is the lower branch of the Lambert W function.

5.3 Approximative equation for w^*

Once the coupling of the neurons in the network is strong enough[1], the value of w at which the V-nullcline is crossed by the trajectory (see section "Regular networks and Dirac synapses" and Figure 5 in the main text) is located in a region where the shape of the nullcline is dominated by the exponential term. Because of this, even quite significant changes in the value of w^* lead to small changes for $V_{NV}(w^*) \approx \ln(w^*)$ as $w^* = n_s b$ is large.
Thus, $V_{NV}(w^*) \approx C$, which is a constant, and, from Eq. S5, we get:

$$V_r e^{-d} + (E_L + I_e - w)(1 - e^{-d}) + \overline{k} Q_s \approx C, \tag{S7}$$

hence

$$w^* \approx E_L + I_e + \frac{V_r e^{-d} + \overline{k} Q_s}{1 - e^{-d}} + C. \tag{S8}$$

6 BURSTING IN HETEROGENEOUS NETWORKS: CONTINUOUS SYNAPSES

6.1 Interspike approximation

As for the Dirac model, we take

$$\begin{aligned} t_s^{(c)}(w) &= \int_{t_A}^{t_B} dt = \int_{V_r}^{V_p} \frac{dV}{-V + e^V + E_L + I_+ - w} \\ &\approx \int_{V_r}^{0} \frac{dV}{E_L - V + I_+ - w} + \int_0^{V_p} \frac{dV}{e^V + E_L + I_+ - w} \end{aligned}$$

Setting $\alpha = E_L + I_+ - w$ and $\beta = e^{V_p} + \alpha/2$, we obtain:

$$t_s^{(c)}(w) \approx \ln\left(\frac{\alpha - V_r}{\alpha}\right) + \frac{1}{\alpha} \ln\left|\frac{(1+\alpha)(2\beta - \alpha)}{2\beta + \alpha}\right| \tag{S9}$$

6.2 Self-consistent equation

The upper bound for w is the value $w^* \approx E_L + I_+ - V_r$ at which spiking does not occur anymore, i.e. when the time between two spikes becomes infinite. As w varies by steps of $\tilde{b}$ for individual neurons, the average interspike during the burst, $\overline{t_s}(w^*)$, can be approximated by integrating the exact expression of $t_s(w)$ on $[w_{min}, w^* - b]$, where the integrand does not diverge. To keep the coherence with the other two models, though the total burst duration of the continuous model must be taken as the sum of the spike times plus the delay necessary for the last spike to arrive:

$$T_B = (n_s - 1)\, \overline{t_s}(w^*) + d \tag{S10}$$

[1] We call "strong bursting" a situation where $n_s b \gg 1$, with n_s the number of spikes emitted by a neuron during the burst and $\tilde{b}$ the spike-triggered adaptation.

where

$$\begin{aligned}\overline{t_s}(w^*) &= \frac{1}{w^* - b - w_{min}} \int_{w_{min}}^{w^*-b} t_s^{(c)}(w) dw \\ &= \frac{1}{w^* - b - w_{min}} \int_{w_{min}}^{w^*-b} \left[\ln\left(\frac{\alpha - V_r}{\alpha}\right) \right. \\ &\quad \left. + \frac{1}{\alpha} \ln\left| \frac{(1+\alpha)(2\beta - \alpha)}{2\beta + \alpha} \right| \right] dw.\end{aligned}$$

In addition, the value of w^* leads to the relation:

$$w^* - w_{min} = I_+ - I_e \tag{S11}$$

From Eq. (S10) and (7) we get

$$d + (n_s - 1)\overline{t_s}(w^*) = \frac{n_s \overline{k} Q_s}{I_+ - I_e}$$

which becomes, using Eq. S11, and $n_s = (w^* - w_{min})/b$:

$$d + \left(\frac{w^* - w_{min}}{b} - 1\right) \overline{t_s}(w^*) = \frac{\overline{k} Q_s}{b}. \tag{S12}$$

which leads to the self-consistent equation (8) in the main text.

Note that for this model to make sense with respect to the previous one, we must be able to define an equivalent non-zero burst duration through the interspike, which means that the number of spikes in the burst must be at least two.

7 A MORE DETAILED MODEL: ALPHA-SHAPED SYNAPTIC CURRENTS

More realistic synapses can be represented using the alpha model which is used in the simulations. For this more complex model, we approximate the shape of the post-synaptic current (PSC) by a rectangular current of duration $4\tau_s$ and amplitude $\frac{\overline{k}ce}{4}$. This allows us to separate the interspike into three segments:

1. the delay $\tilde{d}$ before spike arrival, after which the potential reaches V_d,
2. the PSC duration ($4\tau_s$), where V almost linearly evolves from V_d to V_{int},
3. the "divergence time" until V reaches V_p, noted $T_{div}^{(\alpha)}$[2].

[2] This is of course only valid if $t_s^{(\alpha)}(w)$ is greater than $d + 4\tau_s$, which is not verified for very high coupling or high synaptic timescales.

7.1 Interspike approximation

From the three phases of the interspike described above, we derive

$$t_s^{(\alpha)}(w) = d + 4\tau_s + \underbrace{\int_{V_{int}}^{V_p} \frac{dV}{E_L - V + e^V + I_e - w}}_{T_{div}^{(\alpha)}(w)}. \tag{S13}$$

Setting once more $\alpha = E_L + I_e - V_{int} - w$, $\beta = e^{V_p} + \alpha/2$, $\gamma = e^{V_{int}} + \alpha/2$, leads to

$$V_d \approx V_r e^{-d} + (E_L + I_e - w)(1 - e^{-d}),$$

hence

$$V_{int} \approx V_d + 4\tau_s \left(I_e + \frac{\overline{k}ce}{4} + E_L - V_d - w \right)$$

and

$$T_{div}^{(\alpha)}(w) \approx \begin{cases} \frac{1}{\alpha} \ln \left| \frac{(2\gamma+\alpha)(2\beta-\alpha)}{(2\gamma-\alpha)(2\beta+\alpha)} \right| & \text{if } V_{int} > 0 \\ \ln \left(\frac{\alpha - V_{int}}{\alpha} \right) + \frac{1}{\alpha} \ln \left| \frac{(1+\alpha)(2\beta-\alpha)}{2\beta+\alpha} \right| & \text{if } V_{int} < 0 \end{cases}$$

7.2 Self-consistent equation

As in the other two models, we can easily derive a condition for the termination of the burst as the divergence occurs only if $V_{int}(w) \geq V_{NV}(w)$ i.e. if the potential after the last spike does not manage to get over the nullcline, the equation of the nullcline being given by $V_{NV}(w)$. Setting $\zeta = E_L + I_e - w^*$, the intersection occurs for:

$$V_r e^{-d} \; + \; \zeta(1 - e^{-d}) \; + \; 4\tau_s \left(\frac{\overline{k}ce}{4} - V_r e^{-d} + \zeta - \zeta(1 - e^{-d}) \right) = \zeta \; - \; \mathcal{W}_{-1}\left(-e^{\zeta}\right)$$

thus

$$\zeta e^{-d}(4\tau_s - 1) = V_r(4\tau_s - 1)e^{-d} - \overline{k}ce\tau_s - \mathcal{W}_{-1}\left(-e^{\zeta}\right)$$

Developing the expression of ζ finally leads to the self-consistent equation:

$$w^* \; = \; E_L \; + \; I_e \; - \; V_r + \frac{e^d}{4\tau_s - 1} \left[\mathcal{W}_{-1}\left(-e^{E_L + I_e - w^*}\right) + \overline{k}Q_s \right] \tag{S14}$$

8 EFFECT OF THE LAST SPIKE

Depending on the model used for the synapses and on the delay regarding spike transmission, the network might feel the effect of the last spike after a time $\tilde{d}$ following the end of a burst.

In the case of the continuous synapses, this delay is already taken into account inside T_B, so we consider that the burst ends directly with the last reset. Thus, we end up at 0 on Figure 4B, with $V_{max}^{(c)} = V_r$. This takes a time $t_{final} = 0$.

For the other two models, the last spark of activity is felt after the delay $\tilde{d}$. During this delay, the membrane potential decays linearly – cf. Eq. (S2) – to:

$$V_d = V_l(d) \approx V_r e^{-d} + (E_L + I_e - w)(1 - e^{-d})$$

In the case of the Dirac synapses, the potential then undergoes an instantaneous shift which leads to the final value $V_{max}^{(d)} = V_d + \overline{k}Q_s$ after $t_{final} = d$. For alpha-shaped synapses, the potential subsequently increases over $4\tau_s$ to reach $V_{max}^{(\alpha)} \approx V_d + 4\tau_s \left(I_e + \frac{\overline{k}ce}{4} + E_L - V_d - w_{max} \right)$ after $t_{final} = d + 4\tau_s$.

To be coherent with the description of the dynamics in time, presented on Figure 4A, we will consider that the global time is $t = 0$ after the last spark of activity dies out and $V(0) = V_{max}$.

9 RESTING PERIOD: INTERBURST DYNAMICS

During the whole interburst (from 0 to 4 on Figure 4A), we consider that V is always small compared to $V_{th} = 0$, i.e. that its dynamics is described by Eq. (S2) where $I(t)$ simplifies to $\tilde{I}_e$ for all times in $[0; IBI]$, when the effect of the last spike is no longer felt by the neurons.

Note that all subsequent numbers will refer to the circled points marking the different periods on Figure 4.

After the last spike, the system ends up in a highly excited state with $(V, w) = (V_{max}, w_{max})$ – marked 0. In that region, w follows a very simple dynamics which can, at the leading order, be approximated as an exponential decrease from its peak value until it reaches its minimum:

$$\dot{w} \approx \frac{a(\overline{V} - E_L) - w)}{\tau_w} \tag{S15}$$

This simple equation represents the influence of the average potential $\overline{V} = (V_{min} + V_{max})/2$ felt by w during its decay.

The behavior of V, on the other hand, is slightly more complex and can be divided into three distinct phases: a first, abrupt, decrease of duration T_{down}, followed by a short transition period ending at $T_2 = T_{down} + T_{up}$, then a slow increase until T_I (point 1).
The first period is a rapid decrease dominated by the influence of $w \approx w_{max}$. It is followed by a short period T_{up} (between 1 and 2) where it goes from the V-nullcline to the recovery path (dotted line on Figure 4B).

The resting period can thus be characterized by three values (w_{max}, w_{min} and V_{min}) which strongly influences its duration T_I.

9.1 Decrease of V

In order to solve on $[0, T_{down}]$ ([0; 1] region) and get a good approximation for T_{down}, we linearize the slow current. The linearized expression for w is called w_l, the solution of the linearized equation for V is called V_l.

$$w_l(t) = w_{max} + \lambda t.$$

We determine λ by taking the average value $\langle V \rangle = \frac{1}{2}(V_{max} - V^0_{min})$ in the equation of $\dot{w}$.

$$w_l(t) = w_{max} + \frac{1}{\tau_w}\left(\frac{a}{g_L}\frac{V_{min} + V_{max} - 2E_L}{2} - w_{max}\right) t.$$

This linearized expression for w is substituted in the right-hand side of Eq. (S2), its solution with initial condition V_{max} reads

$$V_l(t) = e^{-t}\left(V_{max} - E_L - I_e + w_{max} - \lambda\right) + \lambda(1 - t) + E_L + I_e - w_{max}$$

with

$$\lambda = \frac{1}{\tau_w}\left(\frac{a}{g_L}\frac{V_{min} + V_r - 2E_L}{2} - w_{max}\right).$$

Then T_{down} is the time when the derivative of V_l vanishes, it is given by

$$T_{down} = -\ln\left(\frac{\lambda}{\lambda - V_{max} + E_L + I_e - w_{max}}\right). \tag{S16}$$

9.2 Transition from refractory to recovery

After the initial decrease of V, the trajectory passes the nullcline and reaches the recovery path (from 1 to 2). During this portion of the dynamics, $\dot{V} \ll \dot{w}$, so the evolution of w can be considered to happen at constant $V = V_{min}$ at zeroth order. We use this approximation for $w \in [w^{(1)}, w^{(2)}]$. Considering the 1st order, V then slowly evolves to reach the value corresponding to $w^{(2)}$ along the recovery path. From a biological standpoint, this can be seen as the point where the persistent currents and the hyperpolarization-activated currents compensate the effect of the spike-driven hyperpolarizing currents.

$$\dot{w} \approx \frac{1}{\tau_w}\left[a(V_{min} - E_L) - w\right]$$

hence, setting $t = 0$ at T_{down}:

$$w(t) \approx a(V_{min} - E_L) + \left[w^{(1)} - a(V_{min} - E_L\right] e^{-t/\tau_w} \tag{S17}$$

Putting the solution back into the equation for V and developing around the nullcline point $(V_{min}, w^{(1)})$, we get:

$$\begin{aligned}\dot{V} &= -(V - E_L) + I_e - w\\ &= -(V - V_{min}) - \left[w^{(1)} - a(V_{min} - E_L)\right]\left(1 - e^{-t/\tau_w}\right)\end{aligned}$$

Changing variables for $V(t) = f(t)e^{-t}$, this leads to:

$$
\begin{aligned}
\dot{f} &= \left[V_{min} - \left(w^{(1)} - a(V_{min} - E_L)\right)\left(1 - e^{-t/\tau_w}\right)\right] \\
\Rightarrow f(t) &= \left[a(V_{min} - E_L) - w^{(1)}\right]\left(\frac{\tau_w}{\tau_w - 1} - 1\right) \\
&\quad + \left[V_{min} + a(V_{min} - E_L) - w^{(1)}\right] e^t \\
&\quad + \frac{\tau_w}{\tau_w - 1}\left[w^{(1)} - a(V_{min} - E_L)\right] e^{\frac{\tau_w - 1}{\tau_w}t}
\end{aligned}
$$

Hence

$$
V(t) = V_{min} + \left[a(V_{min} - E_L) - w^{(1)}\right] \cdot \left[\left(\frac{\tau_w}{\tau_w - 1} - 1\right)e^{-t} - \frac{\tau_w}{\tau_w - 1}e^{-t/\tau_w} - 1\right] \quad \text{(S18)}
$$

9.3 Recovery period

After the transition which follows the initial decrease of V, we enter the recovery period – interval between points 2 and 3. There, w slowly decreases to its minimum value w_{min} while V increases until the first spike of the following burst. The trajectory stays close to the V-nullcline, on a path where the derivative of both V and w have the same magnitude, i.e, neglecting the nonlinear terms:

$$
\frac{\dot{w}}{\dot{V}} \approx \frac{a(V - E_L) - w}{\tau_w(I_e + E_L - V - w)} = \frac{dw_{VN}}{dV} \approx -1
$$

which leads to

$$
w = \frac{1}{1 + \tau_w}\left[(a - \tau_w)(V - E_L) + \tau_w I_e\right] \quad \text{(S19)}
$$

or equivalently

$$
V = E_L + \frac{1}{a - \tau_w}\left[(1 + \tau_w)w - \tau_w I_e\right]
$$

During this period, the evolution of w is therefore driven by

$$
\begin{aligned}
\dot{w} &= \frac{1}{\tau_w}\left[a(V - E_L) - w\right] \\
&= \frac{1}{\tau_w}\left[\frac{a}{a - \tau_w}\left((1 + \tau_w)w - \tau_w I_e\right) - w\right] \\
&= -\frac{1 + a}{\tau_w - a}\left(w - \frac{a}{1 + a}I_e\right)
\end{aligned}
$$

This behavior changes when V reaches $V_{th} = 0$, where the non linear terms become predominant and the dynamics of V, again, becomes much faster than that of w.

Thus, the recovery time is given by:

$$T_R = \frac{\tau_w - a}{1+a} \ln\left(\frac{w^{(2)} - \frac{a}{1+a} I_e}{w_{min} - \frac{a}{1+a} I_e}\right)$$

9.4 Initiation of the burst

After the recovery period, the burst is initiated (point 3 on Figure 4) as the trajectory of the mean-field neuron reaches the minimum of the V-nullcline, i.e. for $w^0_{min} = 1 + I_e + E_L$ and $V = V_{th} = 0$. Once V reaches 0, the first spike of the burst is initiated, which takes

$$T_{fs} = \int_{t=T_I}^{T_I+T_{fs}} dt = \int_{V=0}^{V_{peak}} \frac{dV}{\dot{V}} \approx \int_{V=0}^{V_{peak}} e^{-V} dV \approx 1 \qquad \text{(S20)}$$

REFERENCES

Bottani, S. (1995). Pulse-Coupled Relaxation Oscillators: From Biological Synchronization to Self-Organized Criticality. *Physical Review Letters* 74, 4189–4192. doi:10.1103/PhysRevLett.74.4189

Fox, J. J., Jayaprakash, C., Wang, D., and Campbell, S. R. (2001). Synchronization in relaxation oscillator networks with conduction delays. *Neural computation* 13, 1003–1021. doi:10.1162/08997660151134307

Izhikevich, E. M. (2000). Phase equations for relaxation oscillators. *SIAM Journal on Applied Mathematics* 60, 1789–1804. doi:10.1137/S0036139999351001

Somers, D. and Kopell, N. (1993). Rapid synchronization through fast threshold modulation. *Biological Cybernetics* 68, 393–407. doi:10.1007/BF00198772
